# Renal resistive index-guided mean arterial pressure titration in sepsis: a prospective single-center, single-blind, parallel-group randomized controlled trial

**DOI:** 10.1038/s41467-026-75398-7

**Published:** 2026-07-17

**Authors:** Can Wang, Rong Li, Qiqi Li, Meng Wang, Yan Huo, Yaqi Han, Qian Zhang, Bingqi Lu, Zhenjie Hu, Xiaoting Wang, Yangong Chao, Lixia Liu, the Chinese Critical Ultrasound Study Group (CCUSG), Yan Huo, Qian Zhang, Xiaoting Wang, Yangong Chao, Lixia Liu

**Affiliations:** 1 Department of Critical Care Medicine, the Fourth Hospital of Hebei Medical University, Shijiazhuang, China; 2 Department of Critical Care Medicine, Peking Union Medical College Hospital, Chinese Academy of Medical Sciences and Peking Union Medical College, Beijing, China; 3 Department of Critical Care Medicine, The First Affiliated Hospital of Tsinghua University, Beijing, China

**Keywords:** Nephrology, Clinical trial design, Infectious diseases

## Abstract

In sepsis, optimizing mean arterial pressure (MAP) for organ perfusion remains challenging. We conduct a single-center, single-blind, parallel-group randomized controlled trial enrolling 274 sepsis patients to evaluate whether renal resistive index (RRI)-guided MAP titration (MAP titration group, MTG) reduces 28-day all-cause mortality (primary endpoint) compared with conventional treatment (conventional treatment group, CTG). Post-titration, RRI decreases significantly in the MTG versus CTG (0.61 vs. 0.67, *P* < 0.001). However, 28-day mortality does not differ (25/138 vs. 36/136; RR 0.69, 95% CI 0.44-1.08; *P* = 0.11). These findings suggest that RRI-guided MAP titration improves renal hemodynamics but does not reduce mortality in this single-center trial; multicenter confirmation is warranted. (Chinese Clinical Trial Registry, ChiCTR2200057136, registered 1 March 2022; https://www.chictr.org.cn/showprojEN.html?proj=153154).

## Abstract (web summary)

After initial resuscitation, optimal mean arterial pressure (MAP) targets in sepsis remain uncertain. Here, the authors show that renal resistive index-guided MAP titration improves markers of kidney blood flow but does not significantly lower 28-day mortality versus conventional MAP management.

## Introduction

Sepsis is a life-threatening organ dysfunction caused by a dysregulated host response to infection^1^. Maintaining an appropriate mean arterial pressure (MAP) is crucial for organ perfusion, and current Surviving Sepsis Campaign guidelines recommend an initial target MAP ≥ 65 mmHg for adults with septic shock requiring vasopressors^2^. However, the optimal MAP beyond this minimum threshold remains controversial. The SEPSISPAM trial showed that targeting a higher MAP (80–85 mmHg) reduced renal replacement therapy use in patients with chronic hypertension^3^. However, mortality was not significantly reduced, and the strategy was associated with more arrhythmias. More recent evidence demonstrated increased mortality with routine MAP elevation to 80–85 mmHg^4^. These findings underscore the need for individualized rather than fixed MAP targets.

Organ perfusion is determined by cardiac output, MAP, and organ-specific vascular resistance^5,6^. The kidney, with its high basal blood flow and capacity for autoregulation, is particularly sensitive to perfusion pressure changes. The renal resistive index (RRI), calculated as (peak systolic velocity (PSV)  − end-diastolic velocity (EDV)) / PSV^7–12^, can be assessed noninvasively at the bedside via Doppler ultrasound and reflects intrarenal vascular resistance. An elevated RRI (>0.70) is associated with impaired renal perfusion, acute kidney injury (AKI) development, and increased mortality^13–17^.

Importantly, an inverse relationship between RRI and MAP has been observed in septic shock^13,18,19^. Deruddre et al. demonstrated that increasing MAP from 65 to 75 mmHg significantly reduced RRI^20^, and this relationship appears influenced by chronic hypertension and diabetes^13,19^. The renal autoregulatory plateau is defined by a lower limit of autoregulation (LLA); in sepsis and chronic vascular disease, autoregulation is frequently impaired^8,14,21–25^. These findings suggest that titrating MAP to identify the nadir RRI could approximate the patient’s renal LLA, thereby optimizing perfusion while minimizing vasopressor exposure.

Here, we show that an RRI-guided MAP titration strategy would improve renal perfusion and clinical outcomes. We conduct the RRIMAP-sepsis trial to evaluate whether this individualized approach reduces 28-day all-cause mortality compared with conventional MAP management in sepsis patients requiring vasopressors after initial resuscitation.

## Results

### Patients

**Fig. 1:**
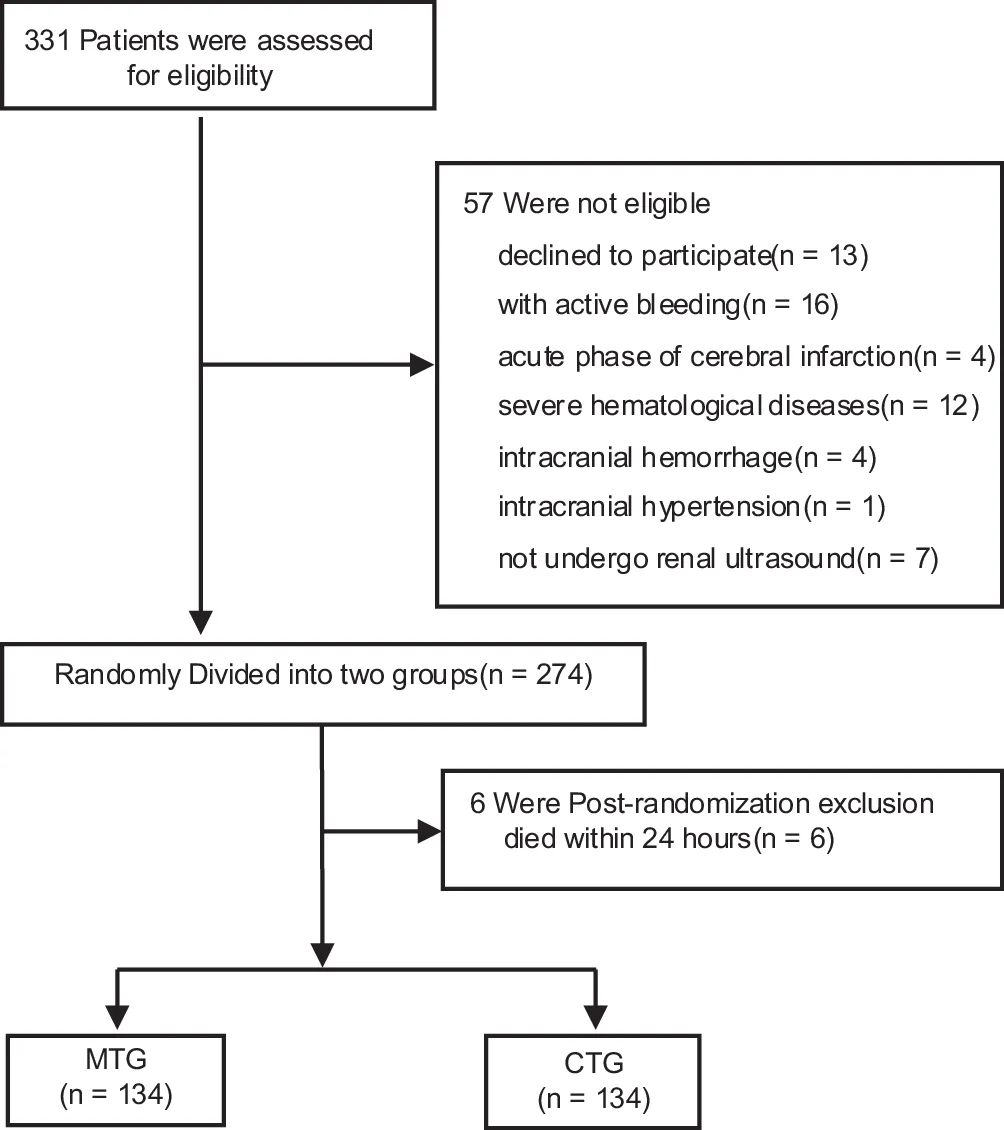
CONSORT diagram of patient flow. This figure shows reasons for exclusion from the study and the numbers of patients included in the analyses. MTG MAP (Mean Arterial Pressure) Titration Group, CTG Conventional Treatment Group.

This trial has been completed. A total of 331 patients were screened for eligibility, of whom 57 were excluded prior to randomization for predefined reasons (Fig. 1). A total of 274 patients underwent randomization and were allocated to the MTG (*n* = 138) or CTG (*n* = 136). Six patients died within 24 h after randomization (4 in the MTG and 2 in the CTG). In accordance with the intention-to-treat principle, all 274 randomized patients were included in the primary analysis and analyzed according to their originally assigned groups, regardless of protocol completion or early death. The remaining 268 patients (MTG, *n* = 134; CTG, *n* = 134), who survived beyond 24 h and completed protocol-directed assessment, constituted the analysis population for secondary outcomes requiring exposure to the intervention. Baseline characteristics, including age, comorbidities, disease severity, AKI status, and hemodynamic parameters, were well balanced between the two groups at enrollment (Table 1). Contrast-enhanced ultrasound (CEUS)-derived microcirculatory parameters, available in the subset of patients who underwent CEUS (MTG, *n* = 81; CTG, *n* = 71), were also comparable between groups at baseline (Table 1). The extent of missing baseline data for all variables is summarized in Supplementary Table S1.

**Table 1:** Baseline characteristics of the two groups

Group (*n*)	MTG (*n* = 134)	CTG (*n* = 134)
Age, years, median (IQR)	67(16)	67(14)
Sex, male, n (%)	93(69.4)	90(67.9)
Height, m, median (IQR)	1.7(0.12)	1.68(0.13)
Weight, kg, median (IQR)	67.5(18.5)	66(15.5)
BMI, kg/m², median (IQR)	23(6)	24(5)
SOFA, median (IQR)	8(5)	8(7)
APACHE II, median (IQR)	19(11)	18(11)
Hypertension, n (%)	62(46.3)	78(58.2)
Diabetes, n (%)	22(16.4)	27(20.1)
Kidney		
AKI, n (%)	48(35.8)	46(34.3)
AKI Stage 1, n (%)	25(18.7)	15(11.2)
AKI Stage 2, n (%)	12(8.9)	16(11.9)
AKI Stage 3, n (%)	11(8.2)	15(11.2)
Duration of AKI, d, median (IQR)	0(1)	0(1)
SCr, μmol/L, median (IQR)	76.2(63.6)	74.7(79.6)
BUN, mmol/L, median (IQR)	8.4(8.0)	8.5(10.78)
RRI, median (IQR)	0.66(0.12)	0.65(0.11)
CKD, n (%)	2(1.5)	2(1.5)
CRRT, n (%)	8(5.9)	13(9.7)
Circulation		
MAP, mmHg, mean (SD)	82 ± 18	83 ± 15
CVP, mmHg, median (IQR)	8(5)	8(5)
CO, L/min, median (IQR)	5.05(2.38)	4.96(2.08)
ScvO_2,_ %, median (IQR)	77.8(13.3)	74.8(11)
Gap, mmHg, median (IQR)	6.1(4.8)	5.7(4.5)
NE dosage, μg/kg/min, median (IQR)	0.20(0.30)	0.20(0.25)
Mechanical ventilation, n (%)	111(82.8)	111(82.8)
P/F, median (IQR)	250.8(191.53)	200(176.25)
bi, median (IQR)	4.78(6.23)	6.18(6.98)
at, median (IQR)	12.69(7.43)	12.41(7.99)
ttp, median (IQR)	32.42(10.99)	29.18(14.47)
pi, median (IQR)	25.53(7.95)	26.45(8.37)
as, mean (SD)	0.62 ± 0.18	0.63 ± 0.19
dt/2, median (IQR)	94.5(20.68)	90.82(22.77)
ds, median (IQR)	-0.12(0.05)	-0.12(0.05)
auc, median (IQR)	3115.48(1449.39)	3038.33(1578.09)
mtt, median (IQR)	81.07(20.74)	79.02(22)
rBV, mean (SD)	20.13 ± 4.35	20.36 ± 6.27
PI, mean (SD)	0.26 ± 0.06	0.26 ± 0.06

Continuous variables are presented as mean ± SD or median (IQR). Source data are provided as a Source Data file.

CEUS parameters: bi: baseline intensity; at: arrival time; ttp: time to peak; pi: peak intensity; as: ascending slope; dt/2: time to half-peak intensity; ds: descending slope; auc: area under the curve; mtt: mean transit time; rBV: relative blood volume (rBV = pi − bi); PI: perfusion index (PI = rBV / MTT).

*MTG* MAP titration group, *CTG* conventional treatment group, *AKI* acute kidney injury, *CKD* chronic kidney disease, *CRRT* continuous renal replacement therapy, *RRI* renal resistive index, *SCr* serum creatinine, *BUN* blood urea nitrogen, *P/F* PaO₂/FiO₂ ratio.

### MAP and RRI

**Fig. 2:**
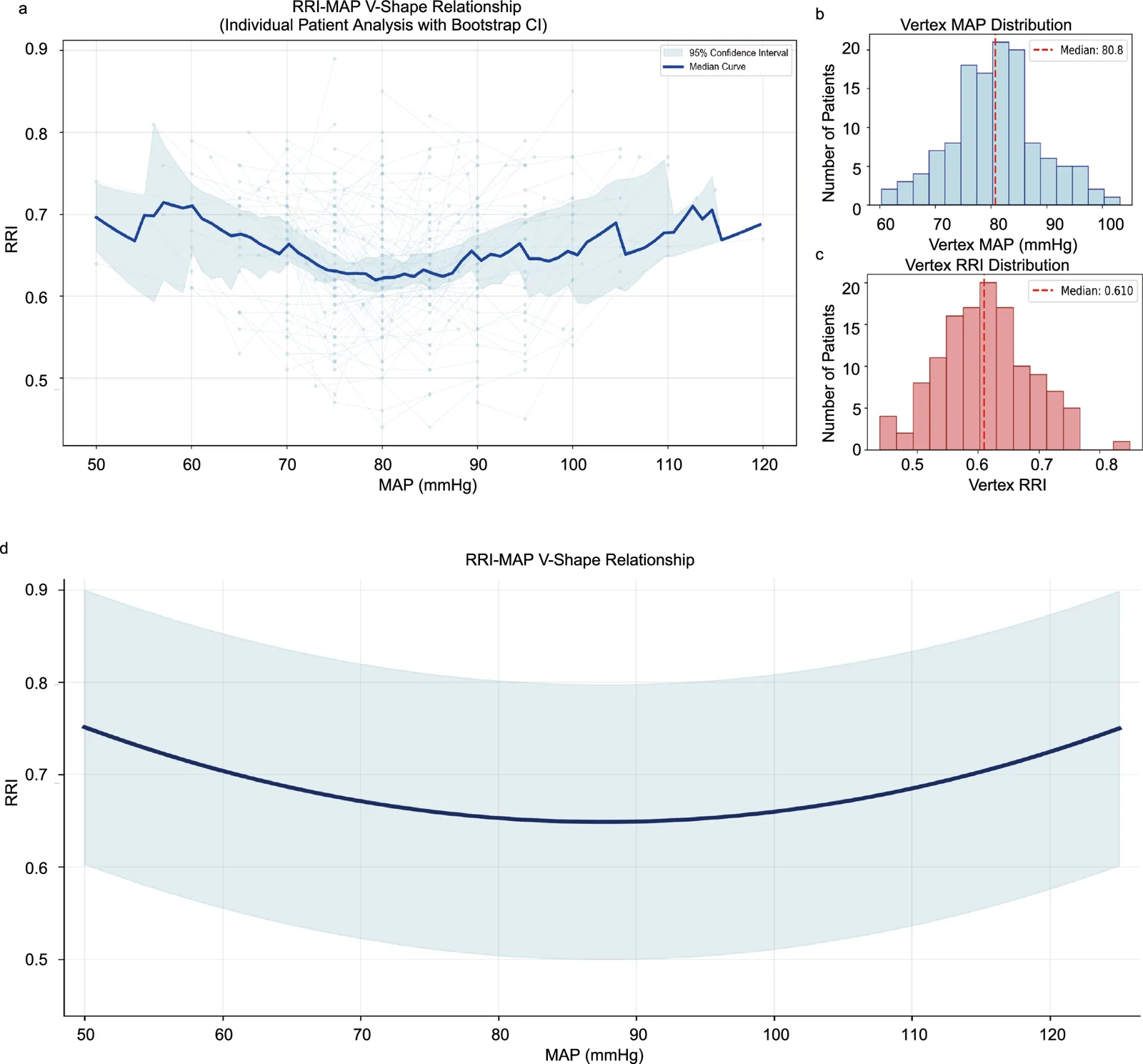
RRI-MAP relationship. **a** Representative per-patient quadratic fits demonstrating that nearly all patients in the MTG exhibited a V-shaped MAP-RRI relationship. Blue line represents the median curve; shaded area represents the 95% confidence interval derived from patient-level cluster bootstrap resampling (B = 1,000). **b** Distribution of vertex MAP values. The predefined criteria included convexity (B2 > 0), an interior vertex (point of lowest RRI), and an RRI increase ≥0.02 within 5-10 mmHg of the vertex. The median (IQR) vertex MAP was 80.8 (76.2–85.3) mmHg. **c** Distribution of vertex RRI values. The median (IQR) vertex RRI was 0.610 (0.563–0.658). **d** Fitted relationship between RRI and MAP during the titration process in all patients. Blue line represents the fitted curve; shaded area represents the 95% confidence interval derived from patient-level cluster bootstrap resampling (B = 1000). Each data point represents an independent biological replicate (individual patient). Source data are provided as a Source Data file.

Inter-observer reproducibility analysis showed good agreement between the two examiners. The mean absolute difference in RRI was 0.011, with a mean bias of −0.001. Bland-Altman analysis demonstrated 95% limits of agreement from −0.029 to 0.027. Based on per-patient quadratic fits, nearly all patients in the MTG group met the criteria for a V-shaped MAP-RRI relationship (Fig. 2a, d). Specifically, the criteria of convexity, an interior vertex, and an RRI increase ≥0.02 within ±5–10 mmHg of the vertex were satisfied in 98.5% of patients (Fig. 2b, c, Fig. S1). For the two patients who did not exhibit a V-shaped change, no clear inflection point was observed, and the target MAP range was conservatively set at 65–70 mmHg (Fig. S2). The mean time-in-target MAP was 89.7% (10.76 h) (see Supplementary Material). RRI measured immediately after titration and at 24 and 48 h remained significantly lower in the MTG (*P* < 0.001; *P* < 0.001, and *P* = 0.04, respectively; Table S2).

#### Exploratory CEUS assessment

CEUS was performed in 81 patients in the MTG and 71 patients in the CTG. Within the MTG, post-titration CEUS parameters were compared with pre-titration values. Peak intensity (pi) increased significantly after titration (pre: 25.95 ± 5.52 vs. post: 28.09 ± 5.95; *P* = 0.031), and the area under the time-intensity curve (auc) also increased (pre: 3092.21 ± 981.36 vs. post: 3467.65 ± 1102.58; *P* = 0.034). These findings suggest that RRI-guided MAP titration may be associated with improved renal cortical microcirculatory perfusion, as reflected by increases in contrast peak intensity and total perfusion area. However, given that CEUS was performed in a subset, only 2 of 11 parameters reached significance, and no between-group differences were detected, these results should be regarded as physiological observations rather than evidence of definitive clinical benefit (Fig. S3, Table S3).

### Outcomes

#### Primary outcome

**Fig. 3:**
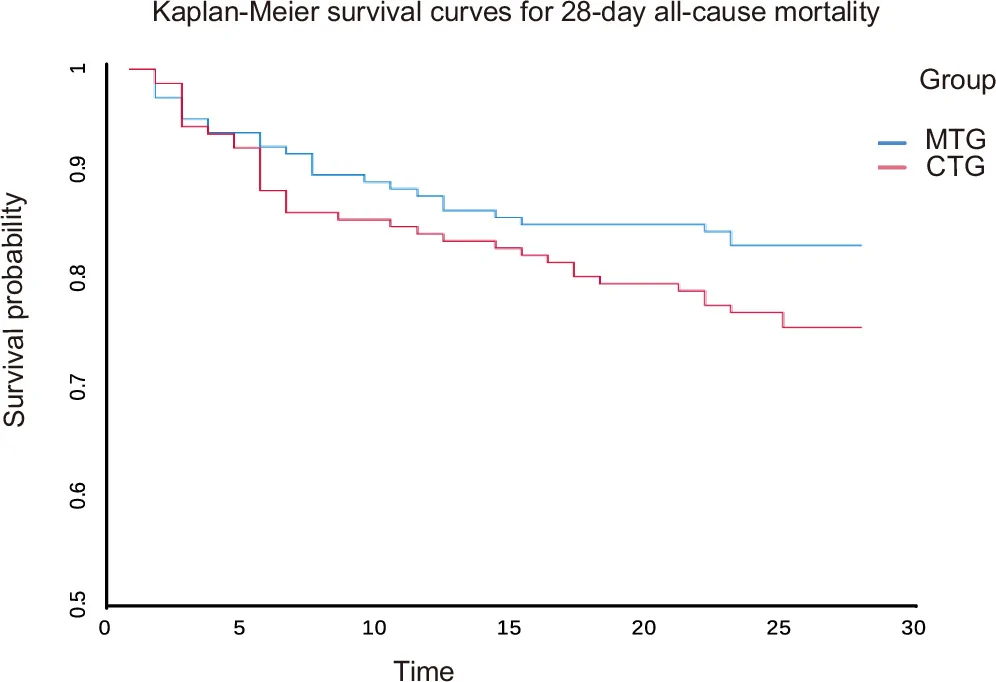
Kaplan-Meier survival curves for 28-day all-cause mortality. MTG (*n* = 138, blue) versus CTG (*n* = 136, red). Groups were compared using a two-sided log-rank test (*P* = 0.107). Cox proportional hazards regression: hazard ratio (HR) 0.69, 95% CI 0.44–1.08. Analysis was performed according to the intention-to-treat principle. Source data are provided as a Source Data file.

The 28-day all-cause mortality was 18.1% (25/138) in the MTG and 26.4% (36/136) in the CTG. The MAP titration strategy did not significantly reduce the 28-day mortality rate among sepsis patients (RR, 0.69, 95% CI, 0.44–1.08; *P* = 0.11) (Table 2). Kaplan-Meier survival curves showed no significant difference in survival distribution between the two groups (*P* = 0.107) (Fig. 3).

**Table 2:** Primary outcomes

	MTG (*n* = 138)	CTG (*n* = 136)	RR (95% CI)	*P value*
Primary Outcome 28-day mortality—no./total no. (%)	25(18.1)	36(26.4)	0.69(0.44–1.08)	0.11

All statistical tests are two-sided. The primary outcome was compared using a chi-squared test. Risk ratio (RR) with 95% confidence interval (CI) is reported. Analysis was performed according to the intention-to-treat (ITT) principle. MTG: MAP (Mean Arterial Pressure) Titration Group (*n* = 138); CTG: Conventional Treatment Group (*n* = 136). Source data are provided as a Source Data file.

#### Secondary outcomes

**Fig. 4:**
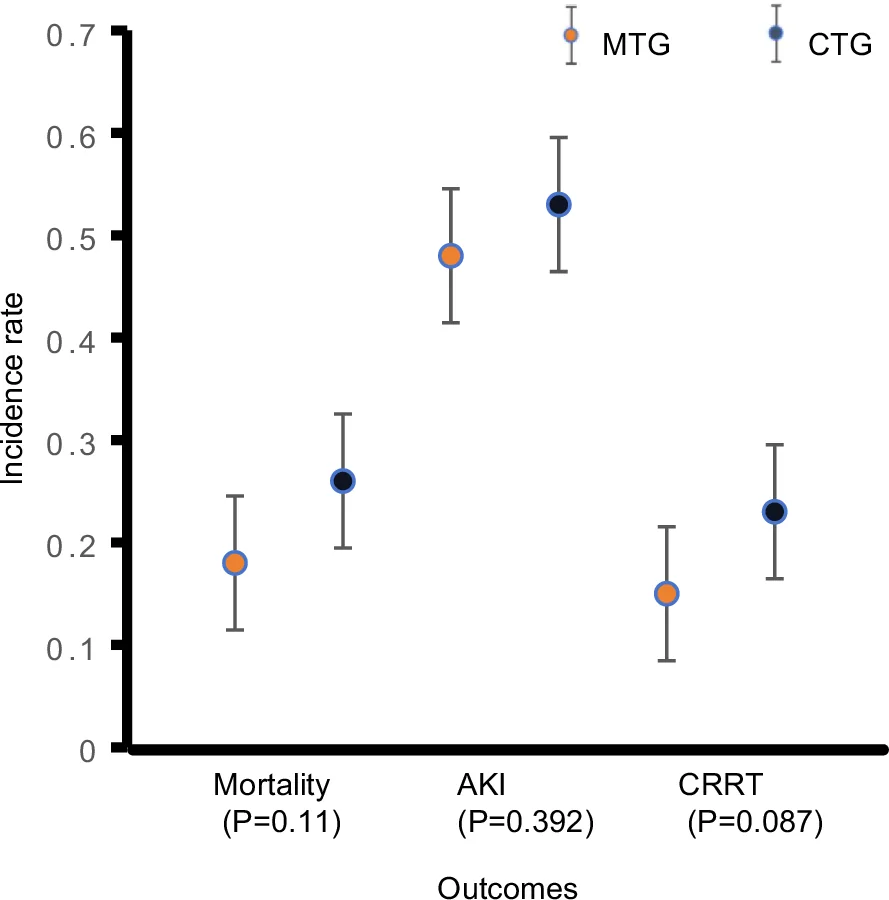
Comparison of clinical outcome incidence rates between MTG and CTG. Data are presented as incidence rates (dots) with 95% confidence intervals (error bars). Mortality (ITT population): MTG *n* = 138, CTG *n* = 136, two-sided chi-squared test, RR = 0.69, 95% CI 0.44–1.08, *P* = 0.11. AKI (modified analysis population): MTG *n* = 134, CTG *n* = 134, two-sided chi-squared test, RR ; 0.901, 95% CI 0.710–1.14, *P* = 0.392. CRRT (modified analysis population): MTG n = 134, CTG n = 134, two-sided chi-squared test, *P* = 0.087. Each dot represents the group-level incidence rate; error bars indicate 95% confidence intervals. Source data are provided as a Source Data file.

AKI stage categories were assigned based on serum creatinine and urine output criteria derived from KDIGO definitions; each patient was classified by the highest stage reached during 28 days into four mutually exclusive categories. The overall incidence of any AKI did not differ between groups (64/134 vs. 71/134; RR, 0.901, 95% CI, 0.710–1.144; *P* = 0.392). The overall distribution across AKI severity stages also did not differ significantly (*χ*²(3) = 6.88, *P* = 0.076). The MTG demonstrated longer ventilator-free days (19 [15] vs. 16 [22]days; *P* = 0.040) and continuous renal replacement therapy (CRRT)-free days (28 [3] vs. 28 [12] days; *P* = 0.012) (Table 3) (Fig. 4).

**Table 3:** Secondary outcomes, clinical results, and serious adverse events

	MTG (*n* = 134)	CTG (*n* = 134)	RR (95% CI)	*P value*
Secondary Outcomes				
28-day incidence of AKI, no./total no. (%)	64(47.7)	71(52.9)	0.901(0.710–1.144)	0.392
Highest AKI stage reached during 28 days, no./total no. (%)				0.076*
No AKI	70(52.3)	63(47.1)		
AKI Stage 1	22(16.4)	23(17.1)		
AKI Stage 2	24(17.9)	15(11.1)		
AKI Stage 3	18(13.4)	33(24.6)		
28-day CRRT, no./total no. (%)	20(14.9)	31(23.1)	0.645(0.388–1.073)	0.087
28-day AKI-free, days	26(12)	25(20)		0.068
28-day CRRT-free, days	28(3)	28(12)		0.012
28-day MV-free, days	19(15)	16(22)		0.040
28-day ICU stay, days	12(15)	14(16)		0.722
Adverse events—no./total no. (%)				
Arrhythmia within one week	56(41.7)	74(55.2)	0.757(0.589–0.973)	0.028
Cerebrovascular accident within one week	10(7.4)	6(4.4)	1.677(0.623–4.456)	0.302
Peripheral ischemia within one week	9(6.7)	12(8.9)	0.750(0.327–1.721)	0.459
Gastrointestinal ischemia within one week	13(9.7)	17(12.6)	0.756(0.387–1.511)	0.438

All statistical tests are two-sided. Categorical variables were compared using chi-squared or Fisher’s exact tests. Continuous variables (28-day AKI-free, CRRT-free, MV-free, and ICU stay days) are presented as median (IQR) and compared using Wilcoxon rank-sum tests. Risk ratios (RR) with 95% confidence intervals (CI) are reported for categorical outcomes. Bonferroni, Holm, and Benjamini-Hochberg corrections were applied for multiple comparisons among secondary endpoints (see Table S6). Analysis was performed in 268 patients who survived beyond 24 h (modified analysis population). For the categorical AKI stage analysis, stages were assigned based on serum creatinine and urine output criteria, while CRRT use was analyzed separately as an organ-support variable. *Chi-squared test for overall distribution across four categories (*χ*²(3) = 6.88, *P* = 0.076). MTG: MAP (Mean Arterial Pressure) Titration Group (*n* = 134). Source data are provided as a Source Data file.

*CTG* conventional treatment group (*n* = 134), *AKI* acute kidney injury, *CRRT* continuous renal replacement therapy, *MV* mechanical ventilation, *ICU* intensive care unit, *IQR *inter quartile range.

### Doses of vasopressor agents, fluid volumes, and other relevant parameters

There were no significant differences between the two groups in the doses of vasopressor agents, fluid balance, diuretic doses, or urine output on days 1, 2, and 3 (Daily norepinephrine (NE) dose, furosemide dose, urine output, and fluid balance data are provided in Supplementary Data 1). Similarly, no statistically significant differences were observed between the groups in oxygenation index, creatinine, blood urea nitrogen, cardiac output (CO), central venous pressure (CVP), or lactate levels before titration, as well as at 48 and 72 h post-titration (Table S2).

### Exploratory subgroup analysis

In the hypertension subgroup, the incidence of AKI at 28 days was significantly lower in the MTG compared with the CTG (7/62 vs. 20/78; RR, 0.440 (95%CI, 0.199–0.974; *P* = 0.033)). Among patients without a history of hypertension, the MTG demonstrated significantly longer ventilator-free days at 28 days compared with the CTG (21 vs 16 days; *P* = 0.031) (Table S4). Subgroup analysis based on AKI status at enrollment indicated that among patients with AKI at baseline, the MTG exhibited significantly longer AKI-free days at 28 days compared with the CTG (20 vs 12 days; *P* = 0.020). For patients without AKI at baseline, the MTG had significantly longer ventilator-free days at 28 days compared with the CTG (21 vs 17 days; *P* = 0.012) (Table S5).

### Adverse Events

During the first week, there was a statistically significant difference in the incidence of arrhythmias with the MTG reducing the incidence of arrhythmias (56/134 vs. 74/134; RR, 0.757 (95%CI, 0.589–0.973; *P* = 0.028)). No significant differences were observed between the two groups in the incidence of cerebrovascular events, peripheral ischemia, and gastrointestinal ischemia (Table 3).

### Multiple comparisons

To address potential type I error due to multiple comparisons among secondary endpoints, we performed sensitivity analyses using three correction methods: Bonferroni, Holm, and Benjamini-Hochberg (FDR). The analyses included ventilator-free days, CRRT-free days, and 7-day arrhythmia risk. Under the conservative Bonferroni correction, only CRRT-free days remained statistically significant (adjusted *P* = 0.048), while other outcomes approached significance. Under the FDR method, all endpoints remained statistically significant. Given that these outcomes were secondary and exploratory, the results should be interpreted cautiously and considered hypothesis-generating (Table S6).

## Discussion

In this single-center randomized trial, an RRI-guided MAP titration strategy did not significantly reduce 28-day mortality compared with conventional MAP management. However, the MTG was associated with improvements in several exploratory secondary outcomes, including CRRT-free days and renal perfusion parameters, suggesting potential physiological benefits of individualized MAP optimization.

After titration, RRI decreased significantly in the MTG and remained lower at 24 and 48 h, despite no group-level differences in MAP. This highlights the individualized nature of the intervention: optimal MAP varied among patients, reinforcing the concept that uniform MAP targets may be insufficient for optimizing organ perfusion. The increase in CRRT-free days, despite a 12-h intervention window, suggests that RRI-guided titration may have improved renal hemodynamics during a critical early period. However, this finding should be interpreted cautiously, as mechanistic data are lacking and alternative explanations—including closer monitoring (Hawthorne effect) or statistical variation—cannot be excluded. Because multiple secondary outcomes were examined, these findings should be considered exploratory and hypothesis-generating.

In our trial, exploratory subgroup analysis suggested that patients with hypertension required higher MAP levels to achieve the lowest RRI, consistent with a rightward shift of the renal pressure-flow curve observed in chronic hypertension^3,18^. Conversely, MAP after titration did not differ between patients with and without baseline AKI, in line with findings by Beloncle et al.^19^ suggesting that AKI does not necessarily alter the MAP-RRI relationship. These observations support a model in which chronic vascular remodeling, rather than acute kidney injury per se, drives shifts in the autoregulatory threshold.

The MTG also demonstrated a lower incidence of 7-day arrhythmias without increased NE use. Unlike strategies employing fixed high MAP targets—which increase catecholamine exposure and arrhythmia risk^3^—our approach individualized MAP according to the MAP-RRI relationship. This distinction may account for the favorable safety profile observed, though confirmation in larger studies is needed.

Notably, the mean MAP in the control group was approximately 80 mmHg, exceeding the 65 mmHg minimum recommended by the SSC^2^. This likely reflects local clinical practice rather than protocol-driven escalation, meaning that our study compares an individualized RRI-guided strategy with a relatively high-MAP standard care approach. This context should be considered when interpreting the findings and may limit generalizability to settings strictly adhering to lower MAP targets.

Our sample size calculation was based on a pilot study suggesting a 14.3% absolute mortality reduction, which we acknowledge may have been optimistic. The observed effect (18.1% vs. 26.4%; RR 0.69 (95% CI 0.44–1.08; *P* = 0.11)) did not reach statistical significance, potentially reflecting both an overestimated pilot effect and limited statistical power. Accordingly, our findings regarding mortality should be interpreted cautiously and considered hypothesis-generating rather than definitive. Larger multicenter trials are needed to determine the true treatment effect.

This study has several limitations. First, as a single-center trial, generalizability is limited; replication of single-center findings in multicenter settings remains a recognized challenge in critical care research^26^. Second, the sample size calculation appears overly optimistic. Third, the intervention duration was limited to 12 h, though early hemodynamic optimization has been associated with improved outcomes^27^. Fourth, baseline creatinine at intensive care unit (ICU) admission may underestimate AKI incidence, but was applied uniformly to both groups, minimizing differential bias. Fifth, other organ perfusion was not simultaneously assessed. Sixth, because treating clinicians were aware of group allocation, outcomes dependent on clinical decision-making (such as ventilator-free days and CRRT-free days) may still be susceptible to performance bias despite the use of standardized protocols. Additionally, the higher-than-guideline MAP in the control group means future studies should compare RRI-guided titration against strict guideline-recommended targets (65–70 mmHg).

In conclusion, the RRIMAP-sepsis strategy did not yield a statistically significant reduction in 28-day mortality among sepsis patients. It was associated with differences in selected secondary outcomes, including prolonged periods free from CRRT and mechanical ventilation (MV) at the 28-day mark, as well as fewer arrhythmias within the first week. Our study further demonstrates the feasibility of implementing RRI-guided MAP titration using bedside renal ultrasonography in the ICU. Larger multicenter trials are required to determine whether this individualized approach translates into clinically meaningful benefit.

## Methods

### Study design

This was a prospective, single-center, randomized controlled trial conducted in the Department of Critical Care Medicine at the Fourth Hospital of Hebei Medical University, a provincial tertiary teaching hospital. The first participant was enrolled on March 2, 2022, and the last on January 28, 2024. The study protocol was approved by the institutional Ethics Committee (Approval No.: 2021KS051), and adhered to the Declaration of Helsinki (1964). Informed consent was obtained from each patient or, for patients unable to consent due to critical illness, from their legally authorized representative (next of kin) before enrollment. The trial was registered with the Chinese Clinical Trial Registry (ChiCTR2200057136) on March 1, 2022, prior to enrollment of the first patient. Details of the study quality assurance procedures: (1) This project established a study quality control team, with the project leader serving as the head of the quality control group. The leader was fully responsible for the design, organization, implementation, and safety of the study. The team consisted of two members: Xiaoting Wang and Yangong Chao. (2) Before the study commenced, all participants underwent training, covering methods, procedures, and data collection. Any questions were directed to the principal investigator and quality control personnel. (3) In case of inconsistent or missing data, immediate queries were made and corrections were implemented. (4) The principal investigator and quality control personnel reviewed screening forms, enrollment forms, follow-up forms, and data consistency and completeness on a monthly basis. Any issues were promptly corrected.

### Study patients

Inclusion criteria: According to the Sepsis-3 International Guidelines, patients diagnosed with sepsis upon ICU admission, who require NE to maintain a MAP ≥ 65 mmHg after Early Goal-Directed Therapy (EGDT) resuscitation, and who are aged ≥18 years. Exclusion Criteria: Pregnant women; Patients expected to undergo emergency surgery within 8 h of sepsis diagnosis; Patients with sepsis complicated by active bleeding; Patients with sepsis complicated by acute cerebral infarction; Patients with sepsis complicated by intracranial hemorrhage and increased intracranial pressure; Patients with sepsis complicated by severe bleeding tendencies, including severe hematologic diseases, hematologic malignancies, and disseminated intravascular coagulation; Patients unable to obtain renal ultrasound images due to surgery, immobilization, or special positioning; Patients in the late stage of disease, expected to die within 24 h of inclusion.

### Randomization, allocation concealment, and blinding

A computer-generated random allocation sequence (1:1 ratio) was created using STATA software by an independent statistician prior to enrollment, who had no role in patient screening, enrollment, or clinical management. After eligibility was confirmed and informed consent was obtained, the enrolling co-investigator assigned participants to study groups according to the predefined randomization sequence. The study employed single-blinding: patients and their families were blinded to group allocation. Treating clinicians were necessarily unblinded because the intervention required bedside ultrasound-guided titration and maintenance of a specific individualized MAP target over 12 h. Outcome assessment and statistical analyses were performed by investigators who were blinded to group allocation.

### RRI-guided MAP titration (RRIMAP-sepsis)

After completion of initial resuscitation according to sepsis guidelines, patients were randomized within 24 h. Two trained ultrasound examiners performed all RRI measurements. To assess measurement reproducibility, a subset of 20 patients underwent duplicate RRI measurements performed independently by the two trained examiners. Inter-observer agreement was evaluated using Bland-Altman analysis. All bedside assessments were performed using a Philips CX50 ultrasound system (curved array probe, 2–5 MHz). Renal hemodynamic parameters, including RRI, were recorded; RRI was calculated as (PSV − EDV) / PSV (Fig. S4). Simultaneously, MAP, heart rate, and the dosage of NE were documented.

#### RRI-guided MAP titration protocol

Patients randomized to the MTG underwent stepwise NE adjustments to identify the individualized MAP associated with the lowest RRI. Each NE change was ±0.02 μg/kg/min, targeting a 5–10 mmHg stepwise change in MAP. After each adjustment, MAP was allowed to stabilize for 5–10 min, then RRI was remeasured. Titration continued until a V-shaped MAP-RRI relationship was observed; the MAP at the nadir RRI was defined as the target MAP. The algorithm did not apply a predefined pathological RRI cutoff. In cases where a reproducible V-shaped relationship was not observed, the target MAP was conservatively set at 65–70 mmHg according to predefined protocol criteria.

**Fig. 5:**
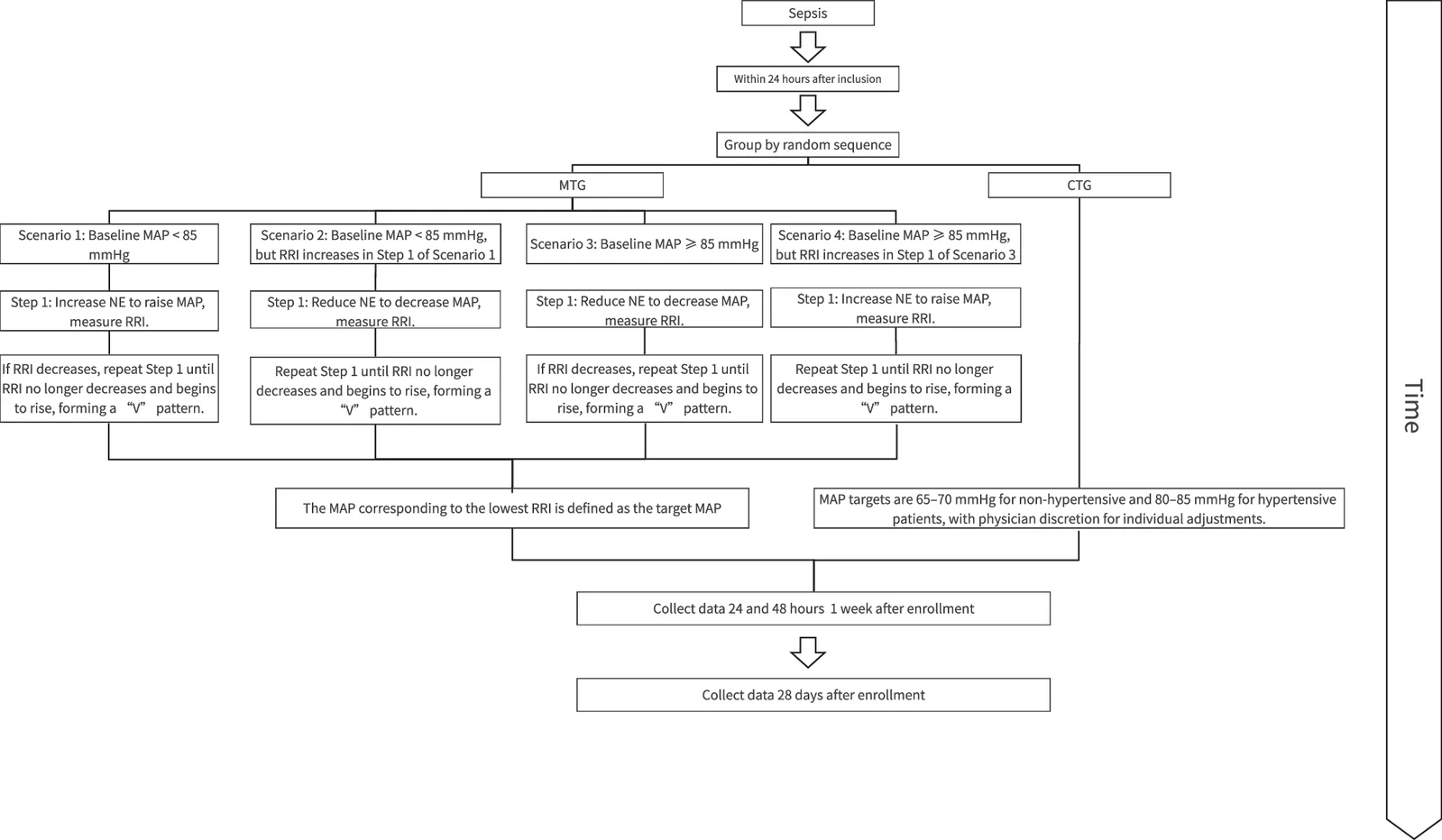
Schematic representation of the RRI-guided MAP titration protocol. Schematic diagram showing the study workflow. Eligible sepsis patients are randomized 1:1 within 24 h of inclusion to MTG or CTG. In the MTG, one of four scenarios is applied based on baseline MAP and the initial RRI response to stepwise NE adjustment (±0.02 μg/kg/min, targeting 5–10 mmHg MAP changes). Titration continues until a V-shaped MAP-RRI relationship is identified, defining the individualized target MAP, which is maintained for 12 h. The CTG follows guideline-recommended MAP targets (65–70 mmHg for non-hypertensive and 80–85 mmHg for hypertensive patients). Clinical and hemodynamic data are collected at 24 h, 48 h, and 1 week after enrollment, and 28-day clinical outcomes were also recorded. The vertical arrow indicates the temporal direction of the protocol. MTG MAP titration group, CTG conventional treatment group, MAP mean arterial pressure, RRI renal resistive index, NE norepinephrine.

Step-by-step algorithm (four scenarios, Fig. 5):

Scenario 1: Baseline MAP < 85 mmHg

- Step 1: Increase NE by 0.02 μg/kg/min to raise MAP in 5–10 mmHg increments. After each adjustment, allow MAP to stabilize for 5–10 min, then measure RRI.
- Step 2: If RRI decreases, repeat Step 1 until RRI no longer decreases and begins to rise, forming a “V” pattern.
- Step 3: The MAP corresponding to the lowest RRI is defined as the target MAP.
  Scenario 2: Baseline MAP < 85 mmHg, but RRI increases in Step 1 of Scenario 1
- Step 1: Reduce NE stepwise by 0.02 μg/kg/min, allowing MAP to decrease in 5–10 mmHg increments. Stabilize and measure RRI at each level.
- Step 2: Repeat Step 1 until RRI decreases and then rises again, forming a “V” pattern.
- Step 3: The MAP at the lowest RRI is defined as the target MAP.
  Scenario 3: Baseline MAP ≥ 85 mmHg
- Step 1: Decrease NE by 0.02 μg/kg/min to lower MAP in 5–10 mmHg increments. After stabilization for 5–10 min, measure RRI.
- Step 2: If RRI decreases, repeat Step 1 until RRI no longer decreases and begins to rise, forming a “V” pattern.
- Step 3: The MAP corresponding to the lowest RRI is defined as the target MAP.
  Scenario 4: Baseline MAP ≥ 85 mmHg, but RRI increases in Step 1 of Scenario 3
- Step 1: Increase NE stepwise by 0.02 μg/kg/min, raising MAP in 5–10 mmHg increments. Stabilize and measure RRI after each adjustment.
- Step 2: Repeat Step 1 until RRI decreases and then begins to rise, forming a “V” pattern.
- Step 3: The MAP at the lowest RRI is defined as the target MAP.

Maintenance phase: Once the individualized target MAP was identified, clinicians maintained MAP within ±5 mmHg of this value for 12 h using NE and fluids as clinically indicated. RRI was measured only during the titration phase, not during the maintenance period (Figs. S5 and S6).

### Protocol rationale

The underlying physiological rationale is that renal vascular resistance may decrease as renal perfusion pressure increases until a plateau is reached, allowing identification of an individualized MAP associated with minimal intrarenal vascular impedance. The titration algorithm was designed based on the following physiological and methodological considerations:

First, the algorithm did not apply a predefined pathological RRI cutoff. Rather, MAP was adjusted to identify the individualized pressure at which the lowest reproducible RRI was observed, hypothesized to correspond to a hemodynamic state with relatively lower intrarenal vascular impedance.

Second, the threshold used to define a meaningful RRI change (ΔRRI ≥ 0.02) was derived from repeatability testing performed under the same blood pressure condition during protocol development. In this assessment, three consecutive RRI measurements showed a short-term measurement fluctuation of approximately 0.02. Measurement repeatability was evaluated using standard agreement analysis methods^28^. Therefore, an RRI change ≥0.02 was considered to exceed expected measurement fluctuation and was interpreted as a directional hemodynamic response during titration rather than measurement noise. RRI was measured three times at each step and averaged to reduce variability.

Third, the stepwise NE adjustment (±0.02 μg/kg/min) and the 5–10-min reassessment interval were selected to allow adequate hemodynamic stabilization after each MAP change and to minimize abrupt perfusion shifts during titration.

The 12-h nocturnal intervention window was chosen to minimize confounding from daytime ICU procedures, to align with the standard nursing shift cycle, and because early hemodynamic optimization within the first 12–24 h of sepsis has been associated with improved outcomes^2,27^.

### Intervention fidelity

Adherence to the intervention was predefined at two levels: (1) completion of the RRI-guided MAP titration algorithm, and (2) maintenance of MAP within ±5 mmHg of the individualized target MAP during the subsequent 12-h intervention window. Titration fidelity required stepwise NE adjustments of ±0.02 μg/kg/min, MAP changes of 5–10 mmHg, a stabilization period of 5–10 min after each adjustment, and triplicate RRI measurements averaged at each pressure level until the MAP associated with the lowest RRI was identified. Maintenance fidelity was quantified as time-in-target MAP during the 12-h intervention period.

Conventional Treatment Group (CTG): target MAP was set at 65–70 mmHg for patients without a history of hypertension and 80–85 mmHg for those with hypertension, in accordance with guideline recommendations^2,3^. Attending physicians were allowed to make discretionary adjustments based on individual patient conditions.

### Outcome assessment

The primary outcome was 28-day all-cause mortality, ascertained by telephone follow-up and hospital record review. Secondary outcomes included AKI incidence and staging, CRRT initiation, AKI-free days, CRRT-free days, ventilator-free days, and ICU length of stay. Adverse events assessed within 7 days included new-onset arrhythmias, cerebrovascular accidents, peripheral ischemia, and gastrointestinal ischemia. As a prespecified exploratory measure, CEUS was performed in a convenience subset of patients in whom logistical conditions permitted (MTG, *n* = 81; CTG, *n* = 71). CEUS was assessed at baseline and after the titrated MAP target had been reached and maintained for 5–10 min under hemodynamically stable conditions.

### Definitions

Baseline MAP was defined as the invasive arterial pressure measured immediately prior to randomization, after completion of initial resuscitation and under stable vasopressor support. All included patients had available baseline MAP data. Baseline MAP was used for descriptive stratification only and did not determine the titration target, which was identified individually based on the MAP-RRI relationship during the intervention. Baseline serum creatinine (SCr) was defined as the first value measured at ICU admission, obtained from the hospital electronic medical record system. AKI was staged according to the 2012 Kidney Disease: Improving Global Outcomes (KDIGO) criteria^29^, and each patient was classified according to the highest AKI stage reached during the 28-day follow-up or before discharge. Ventilator weaning followed a standardized ICU protocol based on daily readiness assessment and spontaneous breathing trials, consistent with ATS/ACCP clinical practice guidelines^30^ for liberation from mechanical ventilation. Patients underwent daily screening for readiness to wean, including: resolution or improvement of the acute cause of respiratory failure, adequate oxygenation (PaO₂/FiO₂ ≥ 150 with PEEP ≤ 8 cmH_2_O), hemodynamic stability without escalating vasopressor support, presence of spontaneous respiratory effort. Patients meeting readiness criteria underwent a spontaneous breathing trial (SBT) lasting 30–120 min. Extubation was performed if the SBT was tolerated without signs of respiratory distress, hemodynamic instability, or excessive work of breathing. CRRT initiation and discontinuation decisions were made by the attending intensivist but followed predefined institutional quality-control criteria applied uniformly to all patients. CRRT initiation criteria: CRRT was initiated when one or more of the following conditions were present: anuria or severe oliguria, hyperkalemia with electrocardiographic changes, severe metabolic acidosis (pH < 7.2), blood urea nitrogen > 84 mg/dL, serum creatinine >5.65 mg/dL (499 μmol/L), refractory fluid overload. CRRT discontinuation criteria: CRRT discontinuation was considered when patients demonstrated: hemodynamic stability without increasing vasopressor requirements, recovery of urine output (>500 mL/day without diuretics), adequate metabolic control, including resolution of severe acidosis, hyperkalemia, and uncontrolled fluid overload. These criteria were applied independently of study group allocation. For the categorical AKI stage analysis in this study, patients were classified according to serum creatinine and urine output criteria, whereas CRRT use was analyzed separately as an organ-support variable. Vasopressor exposure was expressed as NE bitartrate dose. CEUS assessment: Renal CEUS was performed in the intervention group both before and after titration, and once at enrollment in the control group. A Mindray M9 ultrasound system equipped with a broadband convex probe (2–5 MHz) was used, and SonoVue (Bracco, Italy) served as the contrast agent. The region of interest (ROI) was set in the renal cortex. The contrast agent was reconstituted with an equal volume of 0.9% sodium chloride solution and agitated according to manufacturer instructions. After confirming a clear longitudinal view of the kidney in B-mode, 1.2 mL of the contrast agent was injected intravenously, followed by a 10 mL saline flush. This bolus injection produced a rapid wash-in and a slower wash-out phase, allowing for the generation of a time–intensity curve. Renal perfusion parameters were extracted from the curve, including: baseline intensity (bi), arrival time (at), time to peak (ttp), peak intensity (pi), ascending slope (as), time to half-maximum intensity decay (dt/2), descending slope (ds), curve width, area under the curve (auc), mean transit time (mtt), relative blood volume (rBV = pi − bi), and perfusion index (PI = rBV / mtt). Renal perfusion parameters: (1) The curve width and the area under the curve (auc): reflect the total number of contrast agent microbubbles entering or accumulating within the tissue vascular bed over a specific time period, thus indirectly indicating tissue microcirculation perfusion in terms of concentration and unit time. (2) Relative blood volume (rBV): the overall volume of vessels within the ROI, calculated by subtracting thebaseline signal intensity from the peaksignal intensity. (3) Mean transit time (mtt, seconds): the time required for the contrast agent to reach 50% of the maximum intensity signal after destruction. (4) The time to half-peak intensity (dt/2): refers to the point in time when the intensity decreases to half of its peak value after reaching the peak. (5) Descending Slope (ds): refers to the slope between the peak of intensity and the end of perfusion within the ROI. (6) Rise time (rt): peak enhancement time, the time it takes for the contrast agent to reach the maximum intensity signal after destruction. (7) Perfusion index (PI): a composite measurement calculated as rBV/mtt. (8) Arrival slope (as): the reperfusion gradient.

### Sample size calculation

The original protocol (registered in March 2022) planned enrollment of 120 patients based on preliminary clinical estimates. By March 2023, all 120 participants had been enrolled, and an internal review of the observed event rates was conducted to reassess the statistical power of the trial. Using the observed 28-day mortality rates (10.0% in the intervention group and 24.3% in the control group; absolute risk reduction 14.3%), a formal sample size calculation indicated that 268 patients (134 per group) would be required to achieve 80% power with a two-sided α of 0.05, assuming a 10% dropout rate. The protocol amendment was approved by the institutional ethics committee in March 2023, and enrollment under the expanded target began on May 20, 2023.

### Statistical analysis

Analysis populations. The primary outcome (28-day mortality) was analyzed according to the intention-to-treat (ITT) principle, including all 274 randomized patients analyzed in their originally assigned groups, regardless of protocol completion or early death. Protocol deviations were documented but did not lead to exclusion from the primary analysis. Secondary hemodynamic and organ-support outcomes were analyzed in the 268 patients who survived beyond 24 h and received protocol-directed management, as these outcomes required exposure to the intervention. This modified analysis population is explicitly stated wherever secondary outcomes are reported.

MAP-RRI relationship analysis. To explore the patient-level MAP-RRI relationship, per-patient quadratic regression analyses were conducted in the MTG during the titration period. A response was classified as V-shaped if: (a) the fitted quadratic coefficient was positive (B2 > 0); (b) the vertex fell within the observed MAP range; and (c) RRI within ±5–10 mmHg of the vertex was ≥0.02 higher than the minimum value. Patients not meeting these criteria were categorized as flat/indeterminate. Uncertainty was assessed with patient-level cluster bootstrap resampling (B = 1000).

General statistical methods. Continuous variables were expressed as mean ± SD or median (IQR) and compared using *t* tests or Wilcoxon rank-sum tests, as appropriate. Categorical variables were compared using chi-squared or Fisher’s exact tests. AKI severity was analyzed in two ways: first, the overall incidence of any AKI versus no AKI was compared using a chi-squared test; second, patients were classified by the highest KDIGO stage reached during 28 days into four mutually exclusive categories (no AKI, stage 1, stage 2, stage 3), and the distribution between groups was compared using a chi-squared test. Stage-specific counts are reported descriptively. Survival was analyzed by Kaplan-Meier curves with log-rank tests; Cox proportional hazards regression estimated hazard ratios with 95% confidence intervals. A two-sided *P* value < 0.05 was considered statistically significant. To address multiple comparisons among secondary endpoints, Bonferroni, Holm, and Benjamini-Hochberg corrections were applied.

### Reporting summary

Further information on research design is available in the Nature Portfolio Reporting Summary linked to this article.

## Supplementary information

Supplementary Information

Description Of Additional Supplementary File

Supplementary Data 1

Reporting summary

Transparent Peer Review file

## Source data

Source data

## Data Availability

The clinical outcome data and hemodynamic measurement data generated in this study are provided in the Source Data file. The individual de-identified participant data are available under restricted access due to the sensitive nature of clinical data from ICU patients; access can be obtained by submitting a proposal to the corresponding author (Lixia Liu, 47300535@hebmu.edu.cn), signing a data access agreement, and receiving approval. Responses to access requests will be provided within 4 weeks. Data are available immediately following publication with no end date. Source data are provided with this paper.
