# Author Correction: Renal resistive index-guided mean arterial pressure titration in sepsis: a prospective single-center, single-blind, parallel-group randomized controlled trial

**DOI:** 10.1038/s41467-026-79236-8

**Published:** 2026-11-04

**Authors:** Can Wang, Rong Li, Qiqi Li, Meng Wang, Yan Huo, Yaqi Han, Qian Zhang, Bingqi Lu, Zhenjie Hu, Xiaoting Wang, Yangong Chao, Lixia Liu, the Chinese Critical Ultrasound Study Group (CCUSG), Yan Huo, Qian Zhang, Xiaoting Wang, Yangong Chao, Lixia Liu

**Affiliations:** 1 Department of Critical Care Medicine, the Fourth Hospital of Hebei Medical University, Shijiazhuang, China; 2 Department of Critical Care Medicine, Peking Union Medical College Hospital, Chinese Academy of Medical Sciences and Peking Union Medical College, Beijing, China; 3 Department of Critical Care Medicine, The First Affiliated Hospital of Tsinghua University, Beijing, China

**Keywords:** Nephrology, Clinical trial design, Infectious diseases

Correction to: *Nature Communications* https://doi.org/10.1038/s41467-026-75398-7, published online 17 July 2026

In the version of this article initially published, due to a processing error, the Fig. 3 color key labels (currently reading in blue, MTG; in red, CTG) were interchanged. Separately, in the Results “Secondary outcomes” paragraph, in the text now reading “The MTG demonstrated longer ventilator-free days (19 [15] vs. 16 [22] days; *P* = 0.040) and continuous renal replacement therapy (CRRT)-free days (28 [3] vs. 28 [12] days; *P* = 0.012),” 19 [15] vs. 16 [22] days and 28 [3] vs. 28 [12] days both appeared originally as “24 vs. 22 days.” The figure and text have been amended in the HTML and PDF versions of the article.

