# Supplementary material for: Renal resistive index-guided mean arterial pressure titration in sepsis: a prospective single-center, single-blind, parallel-group randomized controlled trial
Source: Nat Commun. 2026 Jul 17;17:8759. doi: 10.1038/s41467-026-75398-7 (PMC13493862; doi:10.1038/s41467-026-75398-7)
Supplement: Supplementary file 1 [file 41467_2026_75398_MOESM1_ESM.pdf]

Renal Resistive Index-Guided Mean Arterial Pressure Titration in Sepsis: A Prospective Single-Center, Single-Blind, Parallel-Group Randomized Controlled Trial (Supplement)

I Missing data handling

All available outcome data were analyzed. Patients without 28-day follow-up information were excluded from the corresponding analyses. No statistical imputation was performed for missing values; the extent of missing data for physiological and laboratory parameters is reported in the TableS1.

The mean time-in-target MAP was 89.7% (10.76 hours).

123 patients (92.2%) maintained MAP within their individualized target range (±5 mmHg) for at least 80% of the 12-hour intervention window (≥10 time points).

11 patients (7.8%) did not achieve the 80% criterion, mainly due to missing data or greater MAP variability.

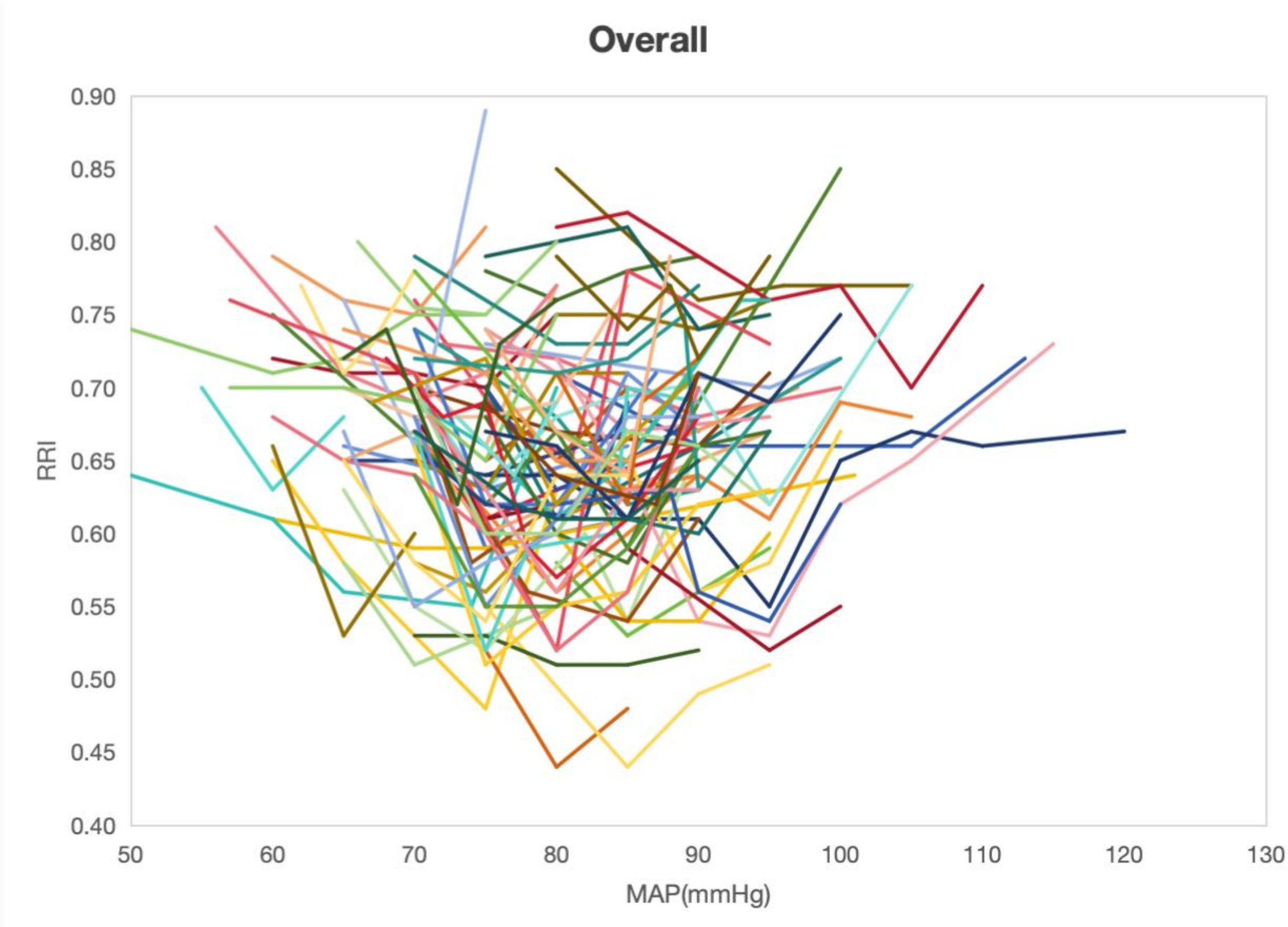

Figure S1. RRI-MAP dynamics across the entire titration protocol. Each line represents the RRI-MAP trajectory of an individual patient in the MTG during the titration process (n=132 patients). The x-axis represents MAP (mmHg); the y-axis represents RRI. Different

colours distinguish individual patients. The overall pattern demonstrates that the majority of patients exhibited a V-shaped RRI-MAP relationship during titration. Source data are provided as a Source Data file.

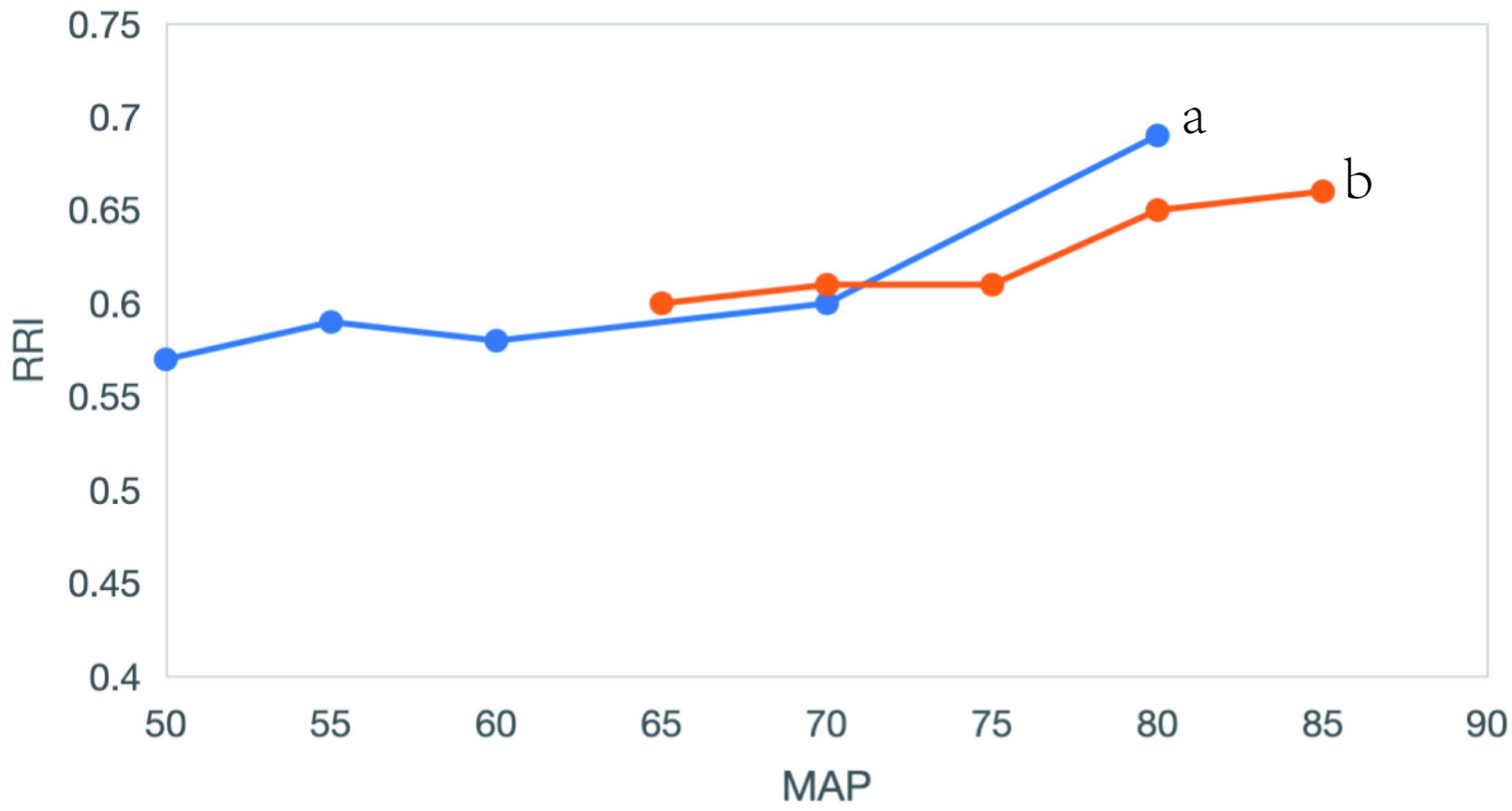

Figure S2. RRI-MAP dynamics during titration in the absence of low inflection points. a Patient one (blue line): RRI increases progressively with MAP from 50 to 80 mmHg without a discernible V-shaped nadir. b Patient two (orange line): RRI increases progressively with MAP from 65 to 85 mmHg without a discernible V-shaped nadir. These patients were classified as flat/indeterminate responders, as they did not meet the predefined V-shape criteria (n=2 representative patients). The x-axis represents MAP (mmHg); the y-axis represents RRI; dots represent individual RRI measurements at each MAP level. Source data are provided as a Source Data file.

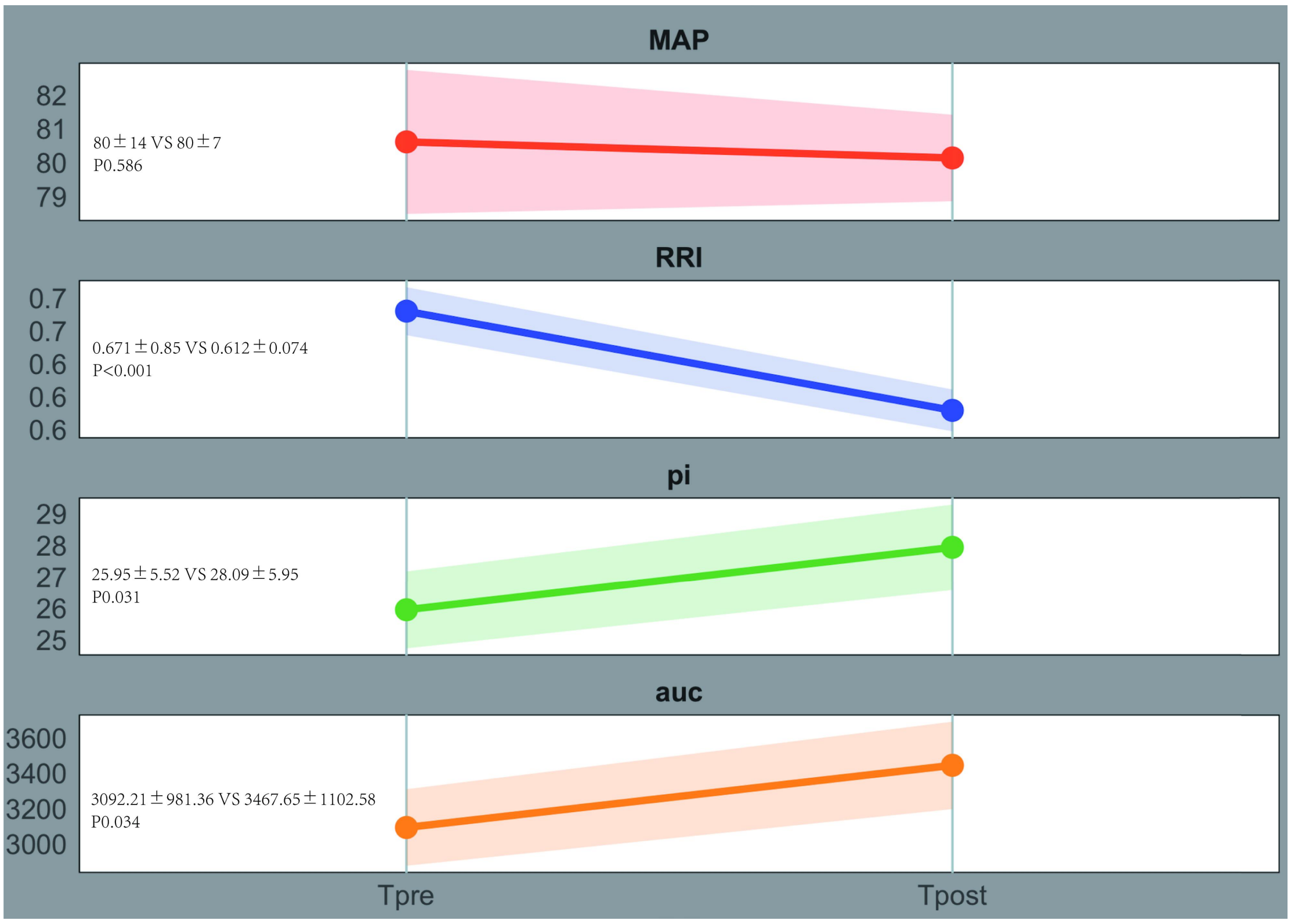

Figure S3. Changes in hemodynamic and renal perfusion parameters pre- and post-titration in the MTG (n=134 patients). Data are presented as mean ± SD with 95% confidence intervals (shaded areas). MAP (red): mean arterial pressure (80 ± 14 vs. 80 ± 7 mmHg, P=0.586, two-sided paired t-test). RRI (blue): renal resistive index (0.671 ± 0.085 vs. 0.612 ± 0.074, P<0.001, two-sided paired t-test). PI (green): perfusion index (25.95 ± 5.52 vs. 28.09 ± 5.95, P=0.031, two-sided paired t-test). AUC (orange): area under the curve from contrast-enhanced ultrasound (3092.21 ± 981.36 vs. 3467.65 ± 1102.58, P=0.034, two-sided paired t-test). The x-axis represents time points (Tpre: pre-titration; Tpost: post-titration). Dots represent mean values; shaded areas represent 95% confidence intervals. Each data point represents an independent biological replicate (individual patient). Source data are provided as a Source Data file.

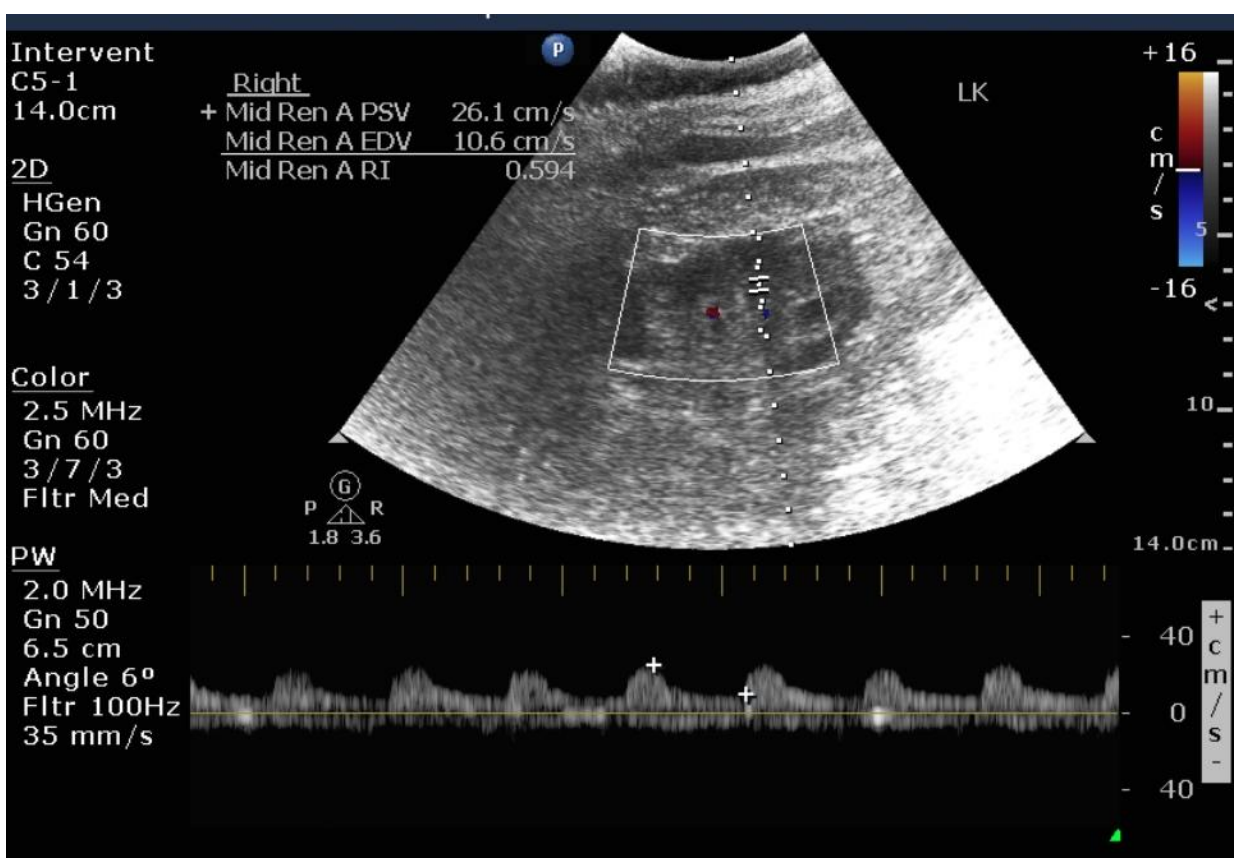

Figure S4. Measurement of renal resistance index

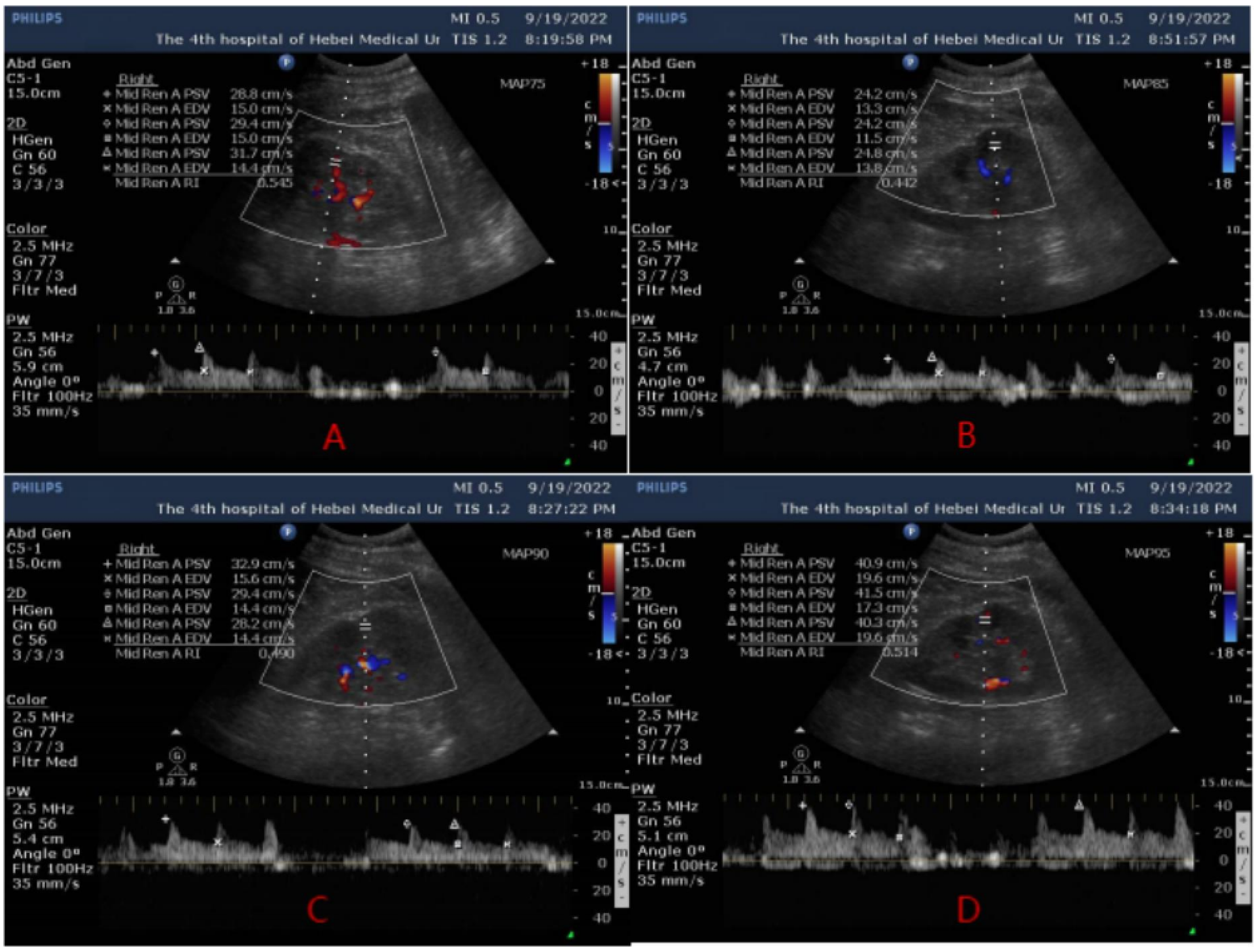

Figure S5. After early EGDT, the patient with sepsis had A MAP of 75mmHg and an RRI=0.545 (A) under NE maintenance. After the first up-regulation of NE, MAP increased to 85mmHg and RRI=0.442 (B). After the second up-regulation of NE, MAP increased to 90mmHg and RRI=0.49 (C). NE was up-regulated for the third time, MAP increased to 95mmHg, RRI=0.514 (D). Combined with RRI results, MAP85-90mmHg was selected as the target MAP.

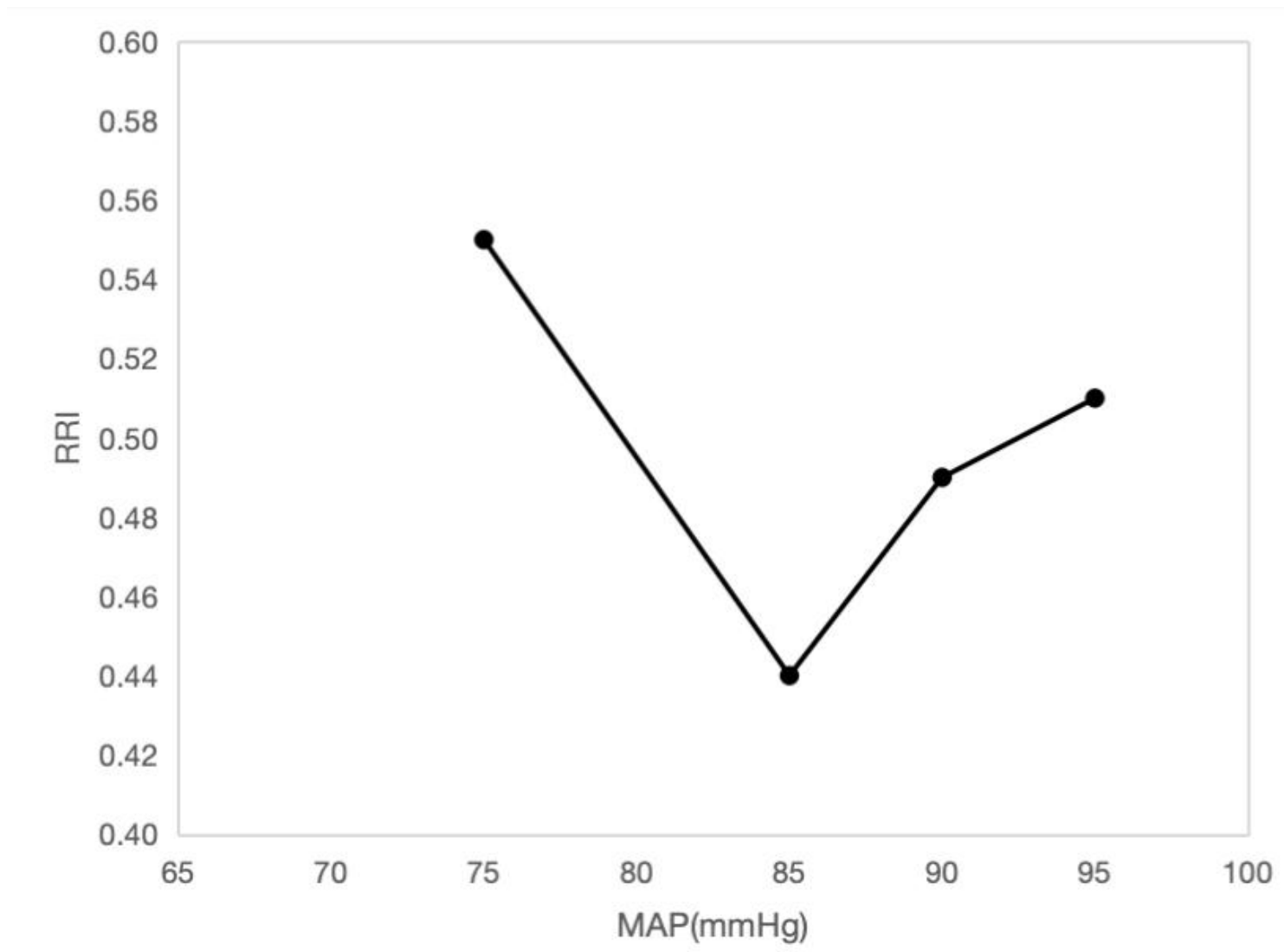

Figure S6. Relationship between RRI and MAP during the titration process in a

representative patient. RRI values were measured at four MAP levels (75, 85, 90, 95 mmHg) during norepinephrine-guided titration, demonstrating a V-shaped pattern with the lowest RRI at MAP 85 mmHg. Data represent a single patient example (n=1 patient). This V-shaped pattern was observed in 1/134 patients in the MTG. The x-axis represents MAP (mmHg); the y-axis represents RRI; black dots represent individual RRI measurements at each MAP level. Source data are provided as a Source Data file.

Table S1 Missing value

	MTG—Missing(n)	CTG—Missing(n)
Tpre CO	14	15
Tpost CO	14	—
T1 CO	19	17
T2 CO	26	27
Tpre CVP	12	20
Tpost CVP	12	—
T1 CVP	13	23
T2 CVP	17	24
T0 Scr	0	0
T1	10	13
T2	12	13
T0 BUN	0	0
T1	10	13
T2	12	13
T0 P/F	6	6
Tpre P/F	2	2
T1 P/F	11	6
T2 P/F	15	16
T0 Lac	4	6
Tpre Lac	2	2
T1 Lac	10	6
T2 Lac	15	16
bi	53	63
at	53	63
ttp	53	63
pi	53	63
as	53	63
dt/2	53	63
ds	53	63
auc	53	63
mtt	53	63
rBV	53	63
PI	53	63

NOTE：MTG：MAP(Mean Arterial Pressure) Titration Group；CTG：Conventional Treatment Group；T0:Admission to the

ICU(Intensive Care Unit); Tpre: Pre–titration; Tpost: Post–titration; T1: 24 hours post–titration; T2: 48 hours post–titration; RRI: Renal Resistance Index; CO: Cardiac Output; MAP: Mean Arterial Pressure; CVP: Central venous pressure; Scr: Serum Creatinine; BUN: Blood Urea Nitrogen; P/F: PaO2/FiO2 ratio; Lac: Lactate; bi: baseline intensity; at: arrival time; ttp: time to peak; pi: peak intensity; as: ascent slope; dt/2: time to half–peak intensity; ds: descent slope; auc: area under the curve; mtt: mean transit time; rBV: relative blood volume (rBV = pi − bi); PI: perfusion index (PI = rBV / mTT).

Table S2 Compared between the two groups pre and post titration

Group	MTG	CTG	*t-value*	*P-value*	F_{group} *P*	F_{time} *P*	$F_{grouptime}$ *P*
n	134	134					
Tpre RRI	0.671±0.085	0.675±0.092	-0.327	0.744	18.450<br><0.001	22.656<br><0.001	23.240<br><0.001
Tpost RRI	0.612±0.074	0.675±0.092	-6.189	<0.001			
T1 RRI	0.644±0.068	0.675±0.079	-3.375	<0.001			
T2 RRI	0.644±0.079	0.672±0.079	-2.876	0.004			
T0 MAP	82±18	83±15	-2.296	0.768	758.912	0.536	0.412
mmHg					0.095	0.586	0.662
Tpre MAP	80±14	82±12	-1.597	0.111			
Tpost MAP	80±7	82±12	-2.194	0.029			
Tpre CO<br>L/min	4.9±1.5	5.0±1.4	-0.564	0.573	2.271<br>0.134	0.345<br>0.005	0.274<br>0.004
Tpost CO	4.8±1.3	5.0±1.5	-0.928	0.355			
T1 CO	4.9±1.3	5.2±1.4	-1.552	0.122			
T2 CO	4.9±1.3	5.2±1.5	-1.452	0.48			
T0 CVP<br>mmHg	8±3	8±3	-1.037	0.301	3.286<br>0.071	3.513<br>0.069	1.396<br>0.028
Tpre CVP	9±2	9±3	-0.533	0.595			
Tpost CVP	9±2	9±3	-1.003	0.317			
T1 CVP	8±3	8±3	-0.496	0.620			
T2 CVP	8±2	10±10	-1.904	0.058			
T0 Scr	104.9±84.2	112.6±94.5	-0.647	0.518	0.477	0.910	1.049
μ mol/L					0.490	0.008	0.009

T1	100.0±58.5	109.8±64.4	-1.195	0.233			
T2	101.9±83.6	102.7±63.0	-0.083	0.934			
T0 BUN	11.24±8.80	19.33±81.00	-1.051	0.295	1.458	0.401	0.441
mmol/L					0.229	0.04	0.04
T1	11.41±7.05	12.42±7.43	-1.045	0.297			
T2	11.41±7.86	12.41±7.16	-0.989	0.324			
T0 P/F	263.09±142.5	237.1±121.1	1.610	0.109	3.600	10.355	0.364
					0.059	<0.001	0.004
Tpre P/F	264.0±113.4	242.0±108.2	1.629	0.104			
T1 P/F	284.5±124.1	274.1±120.5	0.695	0.487			
T2 P/F	296.2±106.8	273.5±86.6	1.910	0.057			
T0 Lac	2.2±1.6	2.1±1.7	0.785	0.450	0.153	13.995	1.086
mmol/L					0.696	0.162	0.015
Tpre Lac	1.9±1.9	1.7±1.7	0.690	0.491			
T1 Lac	1.5±1.2	1.6±1.4	-0.461	0.645			
T2 Lac	1.4±0.7	1.4±1.1	-0.109	0.914			

NOTE：Data are presented as mean ± SD. Between-group comparisons at each time point were performed using two-sided independent samplest-tests. Longitudinal changes were analyzed using repeated measures ANOVA, reporting F statistics for group effect(F_group),time effect(F_time), and group × time interaction(F_group × time). Bonferroni, Holm, and Benjamini-Hochberg corrections were applied for multiple comparisons among secondary endpoints. MTG：MAP(Mean Arterial Pressure) Titration Group(n=134);CTG：Conventional Treatment Group(n=134);T0:Admission to the ICU; Tpre：Pre-titration; Tpost：Post-titration；T1：24hourspost-titration；T2：48hours post-titration；RRI：Renal Resistance Index；CO：Cardiac Output；MAP：MeanArterial Pressure；CVP：Centralvenous pressure；Scr：Serum Creatinine；BUN：Blood Urea Nitrogen；P/F：PaO2/FiO2 ratio；Lac：Lactate.

Table S3 CEUS comparison pre and post titration

	MTG (pre n=81)	MTG (post n=81)	
bi, median (IQR)	4.78(6.23)	6.73(8.10)	0.089
at, median (IQR)	12.69(7.43)	11.57(5.44)	0.195
ttp, mean(SD)	32.67±9.23	32.11±9.04	0.938
pi, mean(SD)	25.95±5.52	28.09±5.95	0.031
as, median (IQR)	0.61(0.24)	0.59(0.20)	0.431
dt/2, median (IQR)	94.5(20.68)	98.22(17.13)	0.490
ds, median (IQR)	-0.12(0.05)	-0.12(0.05)	0.625
auc, mean(SD)	3092.21±981.36	3467.65±1102.58	0.034

mtt, median (IQR)	81.07(20.74)	83.41(22.35)	0.427
rBV, mean(SD)	20.13±4.35	20.65±4.98	0.481

NOTE：Continuous variables are presented as mean ±SD or median (IQR). Comparisons were performed using paired t–tests or Wilcoxon signed–rank tests (two–sided).NOTE：MTG：MAP (Mean Arterial Pressure) Titration Group；at：arrival time；ttp：time to peak；pi：peak intensity；as：ascent slope；dt/2：time to half–peak intensity；ds：descent slope；auc：area under the curve；mtt：mean transit time；rBV：relative blood volume (rBV = pi – bi)；PI：perfusion index (PI = rBV / mTT)；median IQR：Inter Quartile Range；mean±SD：Standard Deviation.

Table S4 Comparison of hypertension with no-hypertension

	Hypertension(n=140)				Non-hypertension(n=128)			
Group	MTG	CTG	RR(95% CI)	P-value	MTG	CTG	RR(95% CI)	P-value
n(%)	62(44.3)	78(55.7)			72(56.2)	56(43.8)		
MAP mean±SD	82±8	83±2		0.590	78±7	67±2		<0.001
RRI median (IQR) /mean±SD	0.61 (0.11)	0.67 (0.12)		0.032	0.61 ±0.07	0.64 ±0.07		0.336
28-mortality n(%)	10(16.1)	21(26.9)	0.599(0.305-1.177)	0.127	11(15.2)	13(23.2)	0.658(0.319-1.356)	0.254
28-day incidence of AKI, no./total no. (%)	32(51.6)	44(56.4)	0.915(0.671-1.248)	0.571	32(44.4)	27(48.2)	0.922(0.634-1.341)	0.671
AKI Stage 1 during 28 days, no./total no. (%)	9(14.5)	14(17.9)	0.809(0.305-1.743)	0.586	13(18.1)	9(16.1)	1.123(0.518-2.438)	0.768
AKI Stage 2 during 28 days, no./total no. (%)	16(25.8)	10(12.8)	2.013(0.983-4.120)	0.050	8(11.1)	5(8.9)	1.244(0.431-3.597)	0.685
AKI Stage 3 during 28 days, no./total no. (%)	7(11.3)	20(25.6)	0.440(0.199-0.974)	0.033	11(15.2)	13(23.2)	0.658(0.319-1.356)	0.254
28-day CRRT n(%)	11(17.7)	22(28.2)	0.542(0.283-1.037)	0.056	9(12.5)	9(16)	0.778(0.331-1.830)	0.564
28-day AKI-free d median (IQR)	24(13)	24(21)		0.422	27(9)	25(20)		0.126
28-day CRRT-free d median (IQR)	28(6)	28(12)		0.138	28(0)	28(8)		0.066
28-day MV-free d median (IQR)	17(18)	16(22)		0.688	21(11)	16(28)		0.031

28-day ICU stay d median (IQR)	12(21)	14(15)	0.737	11(12)	12(19)		0.760

NOTE：All statistical tests are two—sided. Categorical variables were compared using chi—squared or Fisher's exact tests；continuous variables were compared using two—sided independent samplest—tests (for normally distributed data, presented as mean ± SD) or Wilcoxon rank—sum tests (for non—normally distributed data, presented as median (IQR)). Risk ratios (RR) with 95% confidence intervals are reported for categorical outcomes. MTG：MAP (Mean Arterial Pressure) Titration Group；CTG：Conventional Treatment Group；RRI：Renal resistive index；AKI：Acute Kidney Injury；CRRT：Continuous Renal Replacement Therapy；ICU：Intensive Care Unit；IQR：InterQuartile Range；SD：Standard Deviatio.

Table S5 AKI and non-AKI groups at enrollment, Comparison Between the MTG and the CTG

	AKI(n=94)				nnn-AKI(n=174)			
Group	MTG	CTG	RR (95% CI)	P-value	MTG	CTG	RR(95% CI)	P-value
n(%)	48(51.1)	46(48.9)			86(49.4)	88(50.6)		
MAP mean±SD	81±8	84±12		0.141	80±7	82±12		0.099
RRI median (IQR) /mean±SD	0.63 (0.10)	0.69 (0.12)		<0.001	0.60 ±0.08	0.66 ±0.08		<0.001
28-mortality n(%)	8 (16.6)	13 (28.2)	0.590 (0.270-1.290)	0.177	13 (15.1)	21 (23.8)	0.633 (0.339-1.183)	0.146
28-day incidence of AKI, no./total no. (%)	48 (100)	46 (100)	1.000	1.00	19 (22.0)	29 (32.9)	0.670 (0.408-1.101)	0.109
AKI Stage 1 during 28 days, no./total no. (%)	16 (33.3)	15 (32.6)	1.022 (0.574-1.820)	0.940	9 (10.4)	12 (13.6)	0.767 (0.341-1.728)	0.521
AKI Stage 2 during 28 days, no./total no. (%)	19 (39.6)	11 (23.9)	1.655 (0.888-3.086)	0.103	5 (5.8)	4 (4.5)	1.279 (0.355-4.604)	0.706
AKI Stage 3 during 28 days, no./total no. (%)	13 (27.1)	20 (43.5)	0.623 (0.353-1.101)	0.096	5 (5.8)	13 (14.8)	0.394 (0.147-1.057)	0.052
28-day CRRT n(%)	15 (31.2)	19 (41.3)	0.757 (0.440-1.301)	0.311	5 (5.8)	12 (13.6)	0.426 (0.157-1.159)	0.082

28-day AKI-free d	20	12	0.020	28	28	0.156
median (IQR)	(14)	(23)		(7)	(13)	
28-day CRRT-free d	28	25	0.094	28	28	0.041
median (IQR)	(9)	(23)		(0)	(6)	
28-day MV-free d	17	15	0.939	21	17	0.012
median (IQR)	(16)	(19)		(14)	(23)	
28-day ICU stay d	17	16	0.919	10	11	0.736
median (IQR)	(18)	(14)		(12)	(16)	

NOTE：All statistical tests are two–sided. Categorical variables were compared using chi–squared or Fisher's exact tests; continuous variables were compared using two–sided independent samplest–tests (for normally distributed data, presented as mean ± SD) or Wilcoxon rank–sum tests (for non–normally distributed data, presented as median (IQR)). Risk ratios (RR) with 95% confidence intervals are reported for categorical outcomes. MTG：MAP (Mean Arterial Pressure) Titration Group；CTG：Conventional Treatment Group；RRI：Renal resistive index；AKI：Acute Kidney Injury；CRRT：Continuous Renal Replacement Therapy；ICU：Intensive Care Unit；IQR：InterQuartile Range；SD：Standard Deviation.

Table S6 Multiple Comparisons Correction of Secondary Endpoints

Secondary Endpoint	Unadjusted p-value	Bonferroni Adjusted p-value	Holm Adjusted p-value	FDR Adjusted p-value
Ventilator-free days	0.040	0.160	0.060	0.040
CRRT-free days	0.012	0.048	0.048	0.037
7-day arrhythmia risk	0.028	0.112	0.060	0.037

NOTE：All statistical tests are two–sided. P values were adjusted using Bonferroni, Holm, and Benjamini–Hochberg (FDR) corrections for multiple comparisons among secondary endpoints. Unadjusted P values were derived from Wilcoxon rank–sum tests (ventilator–free days, CRRT–free days) and chi–squared test (7–day arrhythmia risk).
