# Supplementary material for: Renal resistive index-guided mean arterial pressure titration in sepsis: a prospective single-center, single-blind, parallel-group randomized controlled trial
Source: Nat Commun. 2026 Jul 17;17:8759. doi: 10.1038/s41467-026-75398-7 (PMC13493862; doi:10.1038/s41467-026-75398-7)
Supplement: Supplementary file 2 [file 41467_2026_75398_MOESM2_ESM.pdf]

Description of Additional Supplementary Files

Supplementary Data 1. Daily norepinephrine dose (mg/day), furosemide dose (mg), urine output (ml), and fluid balance (ml) on days 1–3 for the MAP titration group and the conventional treatment group. Data are presented as median (interquartile range).
