# Supplementary material for: Renal resistive index-guided mean arterial pressure titration in sepsis: a prospective single-center, single-blind, parallel-group randomized controlled trial
Source: Nat Commun. 2026 Jul 17;17:8759. doi: 10.1038/s41467-026-75398-7 (PMC13493862; doi:10.1038/s41467-026-75398-7)
Supplement: Supplementary file 3 [file 41467_2026_75398_MOESM4_ESM.pdf]

nature portfolio

Corresponding author(s): Lixia Liu

Last updated by author(s): May 3, 2026

Reporting Summary

Nature Portfolio wishes to improve the reproducibility of the work that we publish. This form provides structure for consistency and transparency in reporting. For further information on Nature Portfolio policies, see our Editorial Policies and the Editorial Policy Checklist.

Statistics

For all statistical analyses, confirm that the following items are present in the figure legend, table legend, main text, or Methods section.

n/a	Confirmed	
☐	☒	The exact sample size (*n*) for each experimental group/condition, given as a discrete number and unit of measurement
☐	☒	A statement on whether measurements were taken from distinct samples or whether the same sample was measured repeatedly
☐	☒	The statistical test(s) used AND whether they are one- or two-sided<br>*Only common tests should be described solely by name; describe more complex techniques in the Methods section.*
☐	☒	A description of all covariates tested
☐	☒	A description of any assumptions or corrections, such as tests of normality and adjustment for multiple comparisons
☐	☒	A full description of the statistical parameters including central tendency (e.g. means) or other basic estimates (e.g. regression coefficient) AND variation (e.g. standard deviation) or associated estimates of uncertainty (e.g. confidence intervals)
☐	☒	For null hypothesis testing, the test statistic (e.g. *F*, *t*, *r*) with confidence intervals, effect sizes, degrees of freedom and *P* value noted<br>*Give P values as exact values whenever suitable.*
☒	☐	For Bayesian analysis, information on the choice of priors and Markov chain Monte Carlo settings
☐	☒	For hierarchical and complex designs, identification of the appropriate level for tests and full reporting of outcomes
☐	☒	Estimates of effect sizes (e.g. Cohen's *d*, Pearson's *r*), indicating how they were calculated

Our web collection on statistics for biologists contains articles on many of the points above.

Software and code

Policy information about availability of computer code

Data collection	excel
Data analysis	spss27.0 R.4.5.1

For manuscripts utilizing custom algorithms or software that are central to the research but not yet described in published literature, software must be made available to editors and reviewers. We strongly encourage code deposition in a community repository (e.g. GitHub). See the Nature Portfolio guidelines for submitting code & software for further information.

Data

Policy information about availability of data

All manuscripts must include a data availability statement. This statement should provide the following information, where applicable:

- Accession codes, unique identifiers, or web links for publicly available datasets
- A description of any restrictions on data availability
- For clinical datasets or third party data, please ensure that the statement adheres to our policy

Source data are provided with this paper. The clinical outcome data and hemodynamic measurement data generated in this study are provided in the Source Data file. The individual de-identified participant data are available under restricted access due to the sensitive nature of clinical data from ICU patients; access can be obtained by submitting a methodologically sound proposal to the corresponding author (Lixia Liu, 47300535@hebmu.edu.cn), signing a data access agreement, and receiving approval. Responses to access requests will be provided within 4 weeks. Data are available immediately following publication with no end date.

Research involving human participants, their data, or biological material

Policy information about studies with human participants or human data. See also policy information about sex, gender (identity/presentation), and sexual orientation and race, ethnicity and racism.

Reporting on sex and gender	Sex was recorded for all participants at enrollment based on information documented in the electronic medical record. Both male and female patients were eligible for inclusion, and no restrictions were applied based on sex or gender. Because the study was not powered to detect sex-specific treatment effects, no predefined subgroup analyses by sex were performed. However, sex distribution was reported in the baseline characteristics table.
Reporting on race, ethnicity, or other socially relevant groupings	Race and ethnicity were not collected as variables in this study. The trial was conducted at a single center in China with a relatively homogeneous patient population. As a result, analyses stratified by race or ethnicity were not performed.
Population characteristics	Participants were adult patients with sepsis admitted to the intensive care unit at the Fourth Hospital of Hebei Medical University. Eligibility criteria were defined prospectively and included patients who met the Sepsis-3 diagnostic criteria and required vasopressor support. Baseline demographic and clinical characteristics, including age, sex, comorbidities, disease severity, and laboratory parameters, were collected at enrollment and are summarized in Table 1.
Recruitment	Participants were recruited from the intensive care unit of the Fourth Hospital of Hebei Medical University. Eligible patients were screened consecutively according to predefined inclusion and exclusion criteria. Enrollment was conducted between March 2022 and the completion of the target sample size after the protocol amendment. Written informed consent was obtained from patients or their legally authorized representatives prior to enrollment.
Ethics oversight	The study protocol was reviewed and approved by the Institutional Ethics Committee of the Fourth Hospital of Hebei Medical University. The trial was conducted in accordance with the principles of the Declaration of Helsinki and relevant institutional guidelines. Written informed consent was obtained from all participants or their legally authorized representatives prior to enrollment.

Note that full information on the approval of the study protocol must also be provided in the manuscript.

Field-specific reporting

Please select the one below that is the best fit for your research. If you are not sure, read the appropriate sections before making your selection.

☒ Life sciences ☐ Behavioural & social sciences ☐ Ecological, evolutionary & environmental sciences

For a reference copy of the document with all sections, see nature.com/documents/nr-reporting-summary-flat.pdf

Life sciences study design

All studies must disclose on these points even when the disclosure is negative.

Sample size	The original protocol planned enrollment of 120 participants based on preliminary clinical estimates. After completion of this phase, a formal sample size calculation was performed using outcome data from a previously conducted single-center pilot study (n = 60), which observed 28-day mortality rates of 10.0% in the intervention group and 24.3% in the control group. Using a two-sided chi-square test with α = 0.05 and 80% power, the required sample size was estimated to be 268 patients (134 per group), accounting for an anticipated 10% dropout rate. The protocol amendment to expand the target sample size was approved by the institutional ethics committee before additional enrollment began
Data exclusions	No participants were excluded from the primary analysis after randomization. All randomized patients were included in the intention-to-treat analysis according to their originally assigned groups. For secondary outcomes requiring exposure to the intervention, patients who died within 24 hours after randomization were not included in those specific analyses because the intervention could not be completed. These criteria were predefined in the study protocol.
Replication	Each participant in this clinical trial contributed data only once, and outcomes were derived from independent patient observations. As a randomized controlled trial, the study design provides internal validation through comparison between intervention and control groups. No additional experimental replication was performed. Further multicenter studies will be required to confirm the generalizability of these findings.
Randomization	Participants were randomly assigned in a 1:1 ratio to the intervention group or the conventional treatment group. The random allocation sequence was generated using STATA software by an independent statistician who was not involved in patient screening, enrollment, or clinical management. After eligibility confirmation and informed consent, participants were assigned to study groups according to the predefined randomization sequence by the enrolling investigator
Blinding	Participants were blinded to group allocation. Because the intervention involved active MAP titration guided by renal resistive index measurements, treating clinicians were aware of group assignment. Outcome assessors and data analysts were blinded to treatment allocation during statistical analysis

Reporting for specific materials, systems and methods

We require information from authors about some types of materials, experimental systems and methods used in many studies. Here, indicate whether each material, system or method listed is relevant to your study. If you are not sure if a list item applies to your research, read the appropriate section before selecting a response.

Materials & experimental systems

n/a	Involved in the study	
☒	☐	Antibodies
☒	☐	Eukaryotic cell lines
☒	☐	Palaeontology and archaeology
☒	☐	Animals and other organisms
☐	☒	Clinical data
☒	☐	Dual use research of concern
☒	☐	Plants

Methods

n/a	Involved in the study	
☒	☐	ChIP-seq
☒	☐	Flow cytometry
☒	☐	MRI-based neuroimaging

Clinical data

Policy information about clinical studies

All manuscripts should comply with the ICMJE guidelines for publication of clinical research and a completed CONSORT checklist must be included with all submissions.

Clinical trial registration: This clinical trial was registered at the Chinese Clinical Trial Registry (ChiCTR) under the registration number ChiCTR2200057136 on March 1, 2022, prior to patient enrollment.

Study protocol: The study protocol was developed prior to trial initiation and approved by the Institutional Ethics Committee of the Fourth Hospital of Hebei Medical University. The protocol specified the study design, eligibility criteria, interventions, and outcome measures. Amendments to the protocol, including the expansion of the target sample size, were approved by the ethics committee before implementation. The protocol details are described in the Methods section and Supplementary Information.

Data collection: Clinical data were collected prospectively by trained investigators according to the predefined study protocol. Baseline demographic characteristics, clinical variables, laboratory parameters, and outcome data were obtained from the hospital electronic medical record system and standardized case report forms. Data quality was monitored by the study investigators to ensure completeness and accuracy.

Outcomes: The primary outcome of the study was 28-day all-cause mortality. Secondary outcomes included ventilator-free days, CRRT-free days, arrhythmia within 7 days, and changes in hemodynamic and renal perfusion parameters. All outcomes were predefined in the study protocol prior to trial initiation.

Plants

Seed stocks: Not applicable.

Novel plant genotypes: Not applicable.

Authentication: Not applicable.
