# Supplementary material for: Renal resistive index-guided mean arterial pressure titration in sepsis: a prospective single-center, single-blind, parallel-group randomized controlled trial
Source: Nat Commun. 2026 Jul 17;17:8759. doi: 10.1038/s41467-026-75398-7 (PMC13493862; doi:10.1038/s41467-026-75398-7)
Supplement: Supplementary file 4 [file 41467_2026_75398_MOESM5_ESM.pdf]

nature portfolio

# Peer Review File

# Renal Resistive Index-Guided Mean Arterial Pressure Titration in Sepsis: A Prospective Single-Center, Single-Blind, Parallel-Group Randomized Controlled Trial

Corresponding Author: Dr Lixia Liu

**This file contains all reviewer reports in order by version, followed by all author rebuttals in order by version.**

Version 0:

Reviewer comments:

Reviewer #1

(Remarks to the Author)
Results from a small RCT in sepsis management comparing RRI-guided MAP titration with UC.
Primary outcome 28 day all cause mortality.

The study is reported according to CONSORT guidelines and the checklist is provided
Abstract
Acronyms not described (AKI)/ what is conventional management? The results are not reported in full (95% CI not always included, numerators/denominator missing for the risk ratios).
Results
Table 1. the first line, I assume year is a mistake for age (years).
Units missing for some characteristics.
No description for some of the characteristics in the table , for example bi. It would be easier to read with details in table.
Site of infection. Total add up to more than the sample size. Is this because some patients had infection at more than one site?
Table 2. results for primary and secondary outcomes. P-values missing for some outcomes. Please complete, and add these to the text when results are reported in the text.
The outcomes are not clearly labelled, and the footnotes do little to help. I deduce from the text that the line AKI Stage 3 is related to AKI at 28 days, but is this a binary variable, AKI stage 3 / AKI not stage 3? Why is reported 3 times, with dichotomised in 3 different ways? Please label all outcomes clearly in Table 2.
Figure 3. the Kaplan -Meier survival curve. No statistics are presented for the comparison of the survival curves of the two treatment arms. The result is simply stated as no significant difference.
Subgroup analyses
When results are reported in the text please include all data relevant to the result (numbers /total when RR is reported, always include 95% Cis).

Sensitivity analysis.
This section is unclear. Theer is not a baseline difference between the groups in the P/F ratio if the p-value for the comparison is 0.067 as the authors report. Supplement Table 4 is referred to but the title for this table does not indicate what outcome is analysed. The text suggests that it is 28 day mortality, but the unadjusted results are not consistent with previous reporting of the 28 day mortality. Very unclear reporting here and it is difficult to interpret.

A very large number of results are reported, not always clearly labelled and with missing p-values, units missing. Many comparisons are reported, suggesting the authors are searching for statistically significant results.
Generally the report needs to concentrate on the main findings, following the protocol to deliver a clear and consistent message.

Reviewer #2

(Remarks to the Author)
This single-center randomized trial evaluates whether a mean arterial pressure (MAP) titration strategy guided by the renal

resistive index (RRI) reduces 28-day mortality in patients with septic shock. The research question is clinically relevant; however, several methodological issues substantially limit interpretability.

Major comments

Although individualized blood pressure management is a promising concept, the manuscript does not provide an adequate physiological or methodological rationale for the proposed “RRI-guided MAP titration protocol.”Key elements of the protocol appear to be arbitrarily defined, including the RRI cutoff value, the definition of baseline MAP, the magnitude of norepinephrine dose adjustments, and the interval of RRI reassessment. While the authors state that “this threshold was selected based on prior methodological literature on measurement agreement [29] and our pilot reproducibility data, which indicated that intra-observer variability of RRI measurement was within ±0.02,” no quantitative evidence is presented to support the use of this threshold for clinical decision-making in septic shock. The authors should clearly present or cite data linking measurement reproducibility to a clinically meaningful treatment threshold and explain how these considerations informed the titration algorithm.

Because the intervention is complex and treating clinicians were not blinded, intervention fidelity is a critical determinant of internal validity. However, the manuscript does not describe how compliance with the RRI-guided strategy was assessed. The authors should clarify how adherence was operationally defined, how it was measured, and provide quantitative compliance data for the intervention arm. Without such information, it is not possible to interpret the results properly.

The use of baseline MAP as an initial stratification variable raises additional concerns. In patients with septic shock, reliable baseline blood pressure values are frequently unavailable or difficult to define, particularly when vasopressors have already been initiated or when patients are transferred from other settings. The manuscript does not explain how missing or unreliable baseline MAP values were handled. The authors should specify the data source and time window used to define baseline MAP, the proportion of patients with missing data, and the prespecified approach to missingness or uncertainty.

The statistical analysis section does not clearly state whether the primary analysis was conducted according to the intention-to-treat principle or using a per-protocol approach. Please explicitly define the primary analysis population and describe how protocol deviations were handled.

There is an important inconsistency between the original protocol and the manuscript regarding sample size. The protocol reportedly planned enrollment of 120 patients, whereas the sample size calculation in the main text yields a required sample of 268 patients. This discrepancy should be transparently addressed, including when and why the target sample size was changed and whether this modification occurred before or after trial initiation.

Finally, the assumptions underlying the sample size calculation appear overly optimistic. The authors assume a 28-day mortality of 10% in the intervention group compared with 24.3% in the control group, implying a very large absolute risk reduction. Experience in critical care medicine suggests that even multifaceted interventions rarely achieve mortality reductions of this magnitude. The authors should justify this expectation with supporting quantitative data from prior studies or pilot work, or revise the assumptions to more plausible effect sizes.

Minor comments

In the abstract, “$P < 0.05$” should be replaced with the exact p-value.

The method used to determine baseline serum creatinine should be described in detail, including data sources and time windows, as this definition strongly influences the incidence of acute kidney injury.

When reporting norepinephrine doses, the authors should specify that doses refer to norepinephrine base rather than alternative salt formulations.

A recent randomized trial demonstrated increased mortality with routine elevation of MAP targets to 80–85 mmHg (https://link.springer.com/article/10.1007/s00134-025-07910-4), but this study is not cited. Comparison with that trial would strengthen the discussion and better contextualize the present findings.

The sentence “A critical physiological relationship exists between MAP and RRI I n sepsis” should be corrected to “A critical physiological relationship exists between MAP and RRI in sepsis.”

The limitations inherent to single-center trials should be discussed more explicitly, including the well-documented difficulty in replicating promising single-center results in larger multicenter studies. Citation of this manuscript would help clarify this issue (https://link.springer.com/article/10.1186/s13054-023-04755-5).

Reviewer #3

(Remarks to the Author)
Thank you for providing me the opportunity to review this manuscript. Wang and colleagues report a single-center RCT evaluating RRI–guided MAP titration in patients with sepsis requiring vasopressors. The study addresses an important and

unresolved question in sepsis management which is individualization of blood pressure targets beyond a uniform MAP threshold. The physiological rationale is sound, and the trial demonstrates feasibility of bedside US–guided hemodynamic titration. The primary outcome was negative. Some secondary endpoints are positive, however, less so after cadjustment for multiplicity. several methodological and interpretative issues limit the strength of the conclusions. The findings should be considered hypothesis-generating rather than practice-changing.

I have some major comments for the authors
The trial did not demonstrate a statistically significant reduction in 28-day all-cause mortality, which was the prespecified primary endpoint. Nevertheless, the abstract, discussion, and conclusion emphasize reductions in AKI stage 3, organ-support–free days, and arrhythmias, which may overstate the clinical impact of the intervention.The conclusions should be tempered to reflect that the primary endpoint was negative. Secondary and exploratory outcomes should be clearly framed as hypothesis-generating! and language suggesting clinical efficacy should be avoided.

2. A few patients who died shortly after randomization were excluded from the analysis. Excluding patients after randomization—particularly those who die may introduce attrition bias and undermines randomization.I am missing a strict ITT analysis including all randomized patients. I think this should be performed and reported. Or at minimum, a sensitivity analysis including these patients. Please discuss implications of their exclusion.

3. The study was single-blinded, with treating clinicians aware of group assignment. Several secondary outcomes (ventilator-free days, CRRT-free days, ICU length of stay) are susceptible to clinician discretion and therefore to performance and detection bias. This should be explicitly acknowledged for these outcomes. Were any standardized criteria or blinded adjudication used for decisions regarding ventilation or CRRT discontinuation? Was this standardised=?

4. The control group maintained a mean MAP of approx 80 mmHg, which exceeds the Surviving Sepsis Campaign recommendation of ≥65 mmHg. To me, this means: The study compares two relatively high-MAP strategies rather than RRI-guided management versus guideline-based care. Please comment on this and reflect this in the Discuussion. rationale for this higher MAP in the control group should be clarified. I suggest to discuss how this affects generalizability and interpretation of the findings.

5. RRI measurement is very operator dependent, yet all ultrasound assessments were performed by two trained examiners at a single center. No quantitative inter-observer or intra-observer reliability data are provided. Can you provide these? Otherwise, this needs be acknowledged as a limitation.

6. The intervention lasted only 12 hours, whereas hard outcomes (mortality!) were assessed up to 28 days. The causal link between a brief hemodynamic intervention and long-term survival,renal or respiratory outcomes remains uncertain.

7. Numerous secondary and subgroup analyses were performed. After conservative correction methods (Bonferroni), only CRRT-free days remain statistically significant. Emphasis on findings that remain significant only under FDR correction is quite speculative.. I suggest thatsecondary outcomes should be clearly framed as exploratory. avoid drawing strong conclusions from results that are sensitive to the method of multiple-comparison adjustment.

8. Subgroup analyses by hypertension status and baseline AKI were performed, but these analyses appear underpowered and are at risk of false-positive findings. --> I would suggest deleting these or discussing them appropriately as only mechanistc and exploratory.

Minor Comments
1. To my mind, the manuscript would benefit from clearer adherence to CONSORT guidelines reporting.. especially more detailed reporting of allocation concealment and randomization implementation.

2. On several occasions, the authors equate lowest RRI with optimal renal autoregulation. However, RRI is influenced by multiple factors (e.g., pulse pressure, arterial compliance, venous congestion, et cetera) and does not directly measure renal blood flow!

3. CEUS data are presented but appear to be available only in a subset of patients, and the clinical relevance of the observed changes is unclear? Please elaborate and clarify

4. the manuscript is lengthy and contains repeated physiological explanations. I would suggest shortening it

Version 1:

Reviewer comments:

Reviewer #1

(Remarks to the Author)
The authors have provided a thoughtful and constructive response to all my comments, which dealt with the statistical analyses, presentation of results and interpretation, of their clinical trial.
Quantitative results are presented with all relevant details when reported in the text.

Table 1 . Omissions have been corrected. The table now stands as a detailed report without the need to refer to a large number of footnotes.
Table 2. and Figure 3. Further detail for results have been added.
The article is now focused on the main objective, the reporting of the primary outcome. The sensitivity analyses and the results for a large number of exploratory outcomes which added little and were often difficult to interpret have been omitted.
Introduction
Please ensure all acronyms defined at first use (PSV, EDV on page 2 is not defined until page 4).
Secondary outcomes - AKI stage
The incidence of stage 3 AKI at 28 days is reported
“Compared to the CTG, the MTG had a lower incidence of stage 3 AKI at 28 days (18/134 vs. 33/134 RR, 0.545 (95%CI, 0.324-0.919; P=0.020))”
But there is no explanation that this is stage 3 AKI compared with stage1 plus stage 2 AKI plus no AKI. (This is also reported in the abstract without a clear explanation).
It would be preferable and clearer to either analyse and report either the total incidence of AKI compared with no AKI, or the assortment of the patients between the 3 stages of AKI and no AKI.
Table 3 The incidence of AKI is correctly analysed and reported (there is no statistically significant difference between the two groups) but the further analysis of AKI is incorrect. The 4 classes AKI stage1, AKI stage2, AKIstage3 and no AKI are not independent. The correct analysis is a CHI2 (3) for assortment between the 4 classes within the 2 treatment groups. (CHI2 (3) = 6.88, p=0.08). There is no statistically significant difference in how the patients are assorted between the 4 classes.
Figure 4 the data showing individual stages of AKI should be removed.

Reviewer #2

(Remarks to the Author)
After reading the reply letter and revised manuscript, I still have several concerns.

1. In Table 3, stage 3 AKI occurred in 18 patients of the intervention arm but CRRT was performed in 20 patients. Given that receipt of CRRT is considered stage 3 AKI according to the KDIGO guidelines, the authors should clarify this inconsistency.

2. Use of the serum creatinine value at ICU admission as the baseline value typically underestimates the rate of AKI properly since in septic patients such values are already elevated compared to the true baseline value.

Reviewer #3

(Remarks to the Author)
The authors adequately responded to my remarks and I think the manuscript's quality is strongly improved. The authors should be appaluded for this study and I recommend publishing it.

Version 2:

Reviewer comments:

Reviewer #1

(Remarks to the Author)
The authors have provided a detailed and considered response to my final comments concerning the results for the AKI grading. These are now reported accurately and with clarity. I have only reviewed statistical analyses and results and I recommend publishing this study which has been carried out to a high standard.

Reviewer #2

(Remarks to the Author)
The authors have appropriately addressed my previous comments. I have no further comments.

**Open Access** This Peer Review File is licensed under a Creative Commons Attribution 4.0 International License, which permits use, sharing, adaptation, distribution and reproduction in any medium or format, as long as you give appropriate credit to the original author(s) and the source, provide a link to the Creative Commons license, and indicate if changes were made.
In cases where reviewers are anonymous, credit should be given to 'Anonymous Referee' and the source.
The images or other third party material in this Peer Review File are included in the article's Creative Commons license, unless indicated otherwise in a credit line to the material. If material is not included in the article's Creative Commons license and your intended use is not permitted by statutory regulation or exceeds the permitted use, you will need to obtain permission directly from the copyright holder.
To view a copy of this license, visit https://creativecommons.org/licenses/by/4.0/

REVIEWER COMMENTS

Reviewer #1 (Remarks to the Author):

Results from a small RCT in sepsis management comparing RRI-guided MAP titration with UC. Primary outcome 28 day all cause mortality.

The study is reported according to CONSORT guidelines and the checklist is provided

**Abstract**

**Acronyms not described (AKI)/ what is conventional management? The results are not reported in full (95% CI not always included, numerators/denominator missing for the risk ratios).**

Response: We thank the reviewer for these helpful comments. First, we have revised the Abstract to define all abbreviations at first mention, including acute kidney injury (AKI). Second, we have clarified the definition of "conventional management" in the Abstract and Methods, specifying that MAP in the control group was managed according to standard care based on current guidelines and clinician judgment, targeting a MAP ⩾65 mmHg. Third, we have revised the Abstract to report results more completely by providing numerators and denominators for risk ratios, as well as corresponding 95% confidence intervals and exact P values where applicable.

**Results**

**Table 1. the first line, I assume year is a mistake for age (years).**

**Units missing for some characteristics.**

**No description for some of the characteristics in the table , for example bi. It would be easier to read with details in table.**

**Site of infection. Total add up to more than the sample size. Is this because some patients had infection at more than one site?**

Response: We thank the reviewer for these helpful comments regarding Table 1 and have revised the table accordingly.First, the variable previously labeled as "year" has been corrected to "Age, years" to avoid ambiguity. Second, we have carefully reviewed Table 1 and ensured that units are now explicitly provided for all continuous variables, together with their corresponding statistical descriptors (median [IQR] or mean ± SD), in line with reporting standards. Third, we have added clear descriptions and definitions for all abbreviated parameters, including contrast-enhanced ultrasound–derived variables (e.g., bi, at, ttp, pi, auc, mtt, rBV, PI), which are now fully explained in the table footnotes with corresponding units where applicable, thereby improving readability and interpretability. Finally, regarding the site of infection, we have clarified in the table footnote that the total number exceeds the sample size because some patients had infections at more than one anatomical site.

**Table 2. results for primary and secondary outcomes. P-values missing for some outcomes. Please complete, and add these to the text when results are reported in the text.**

Response: We thank the reviewer for this comment. We have systematically reviewed Table 2 and verified that P-values are now provided for all primary outcomes, secondary outcomes, and adverse events without exception. Specifically, P-values have been added for outcomes where

they were previously missing, including gastrointestinal ischemia (P = 0.438). In addition, all reported P-values have been cross-checked against the corresponding Results text to ensure consistency. Where results are reported in the text, the corresponding P-values, effect estimates, and 95% confidence intervals are now included.

**The outcomes are not clearly labelled, and the footnotes do little to help. I deduce from the text that the line AKI Stage 3 is related to AKI at 28 days, but is this a binary variable, AKI stage 3 / AKI not stage 3? Why is reported 3 times, with dichotomised in 3 different ways? Please label all outcomes clearly in Table 2.**

Response: We thank the reviewer for this comment. We have revised Table 2 to clearly specify that AKI staging was assessed at 28 days according to KDIGO criteria, and that stage 1–3 incidences are reported relative to the total study population. The row labels and table footnotes have been updated accordingly to avoid ambiguity.

**Figure 3. the Kaplan -Meier survival curve. No statistics are presented for the comparison of the survival curves of the two treatment arms. The result is simply stated as no significant difference.**

Response: We thank the reviewer for this comment. We have now added the log-rank test P value to Figure 3 and have updated the Results section accordingly to report the statistical comparison between the two groups.

**Subgroup analyses**

**When results are reported in the text please include all data relevant to the result (numbers /total when RR is reported, always include 95% Cis).**

Response: We thank the reviewer for this comment. We have revised all subgroup analyses to include complete reporting. Specifically: (1) Numerators and denominators are now provided for each group when risk ratios are reported (e.g., AKI stage 3 in hypertension subgroup:RR 0.440, 95% CI 0.199–0.974, P = 0.033). (2) For continuous outcomes such as ventilator-free days and AKI-free days, we now report median values with median differences and corresponding 95% confidence intervals. (3) Subgroup sample sizes are explicitly stated. (4) P-values have been added to assess treatment-effect modification. (5) These subgroup analyses are now clearly framed as exploratory and hypothesis-generating, as they were not powered for subgroup comparisons. In addition, the “Renal Autoregulation Assessment” subsection—which contained within-arm observational comparisons rather than between-arm treatment-effect analyses—has been removed to reduce the number of comparisons in the main text and focus on the primary and secondary findings.

**Sensitivity analysis.**

**This section is unclear. Theer is not a baseline difference between the groups in the P/F ratio if the p-value for the comparison is 0.067 as the authors report. Supplement Table 4 is referred to but the title for this table does not indicate what outcome is analysed. The text suggests that it is 28 day mortality, but the unadjusted results are not consistent with previous reporting of the 28 day mortality. Very unclear reporting here and it is difficult to interpret.**

Response: We thank the reviewer for this careful observation. We agree that the presentation of

the sensitivity analysis was unclear and may have been misleading. In particular, our wording was imprecise in referring to “baseline differences,” since the between-group difference in P/F ratio was not statistically significant (p=0.067), and the title/content of Supplementary Table 4 did not clearly identify the analyzed outcome. We also acknowledge that the reporting of the unadjusted estimate in this section was insufficiently clear in relation to the main 28-day mortality results.
Because this analysis was exploratory, was not prespecified, and does not materially affect the interpretation of the primary trial findings, we have removed the sensitivity analysis and the corresponding Supplementary Table 4 from the revised manuscript. We have also deleted the related text from the Results and Discussion sections to avoid confusion and to keep the focus on the primary and prespecified secondary outcomes.

**A very large number of results are reported, not always clearly labelled and with missing p-values, units missing. Many comparisons are reported, suggesting the authors are searching for statistically significant results. Generally the report needs to concentrate on the main findings, following the protocol to deliver a clear and consistent message.**
Response: We have restructured the Results section to focus on the primary endpoint and prespecified secondary outcomes. Specifically, the previously reported sensitivity analysis has been removed. The “Renal Autoregulation Assessment” subsection, which contained within-arm observational comparisons, has been removed. All reported outcomes are now clearly labeled as primary, prespecified secondary, or exploratory, with corresponding P-values, effect estimates, and 95% confidence intervals. Multiple comparison corrections (Bonferroni, Holm, Benjamini–Hochberg) are provided for all secondary endpoints..

Reviewer #2 (Remarks to the Author):

This single-center randomized trial evaluates whether a mean arterial pressure (MAP) titration strategy guided by the renal resistive index (RRI) reduces 28-day mortality in patients with septic shock. The research question is clinically relevant; however, several methodological issues substantially limit interpretability.

Major comments

**Although individualized blood pressure management is a promising concept, the manuscript does not provide an adequate physiological or methodological rationale for the proposed “RRI-guided MAP titration protocol.” Key elements of the protocol appear to be arbitrarily defined, including the RRI cutoff value, the definition of baseline MAP, the magnitude of norepinephrine dose adjustments, and the interval of RRI reassessment. While the authors state that “this threshold was selected based on prior methodological literature on measurement agreement [29] and our pilot reproducibility data, which indicated that intra-observer variability of RRI measurement was within ±0.02,” no quantitative evidence is presented to support the use of this threshold for clinical decision-making in septic shock. The**

**authors should clearly present or cite data linking measurement reproducibility to a clinically meaningful treatment threshold and explain how these considerations informed the titration algorithm.**

Response: We thank the reviewer for this important methodological comment. We agree that the physiological and methodological rationale for the RRI-guided MAP titration protocol required clearer explanation. In the revised manuscript, we have added a dedicated "Protocol Rationale" subsection in the Methods to clarify how the main elements of the titration algorithm were determined.

First, we clarify that the protocol does not rely on a fixed pathological RRI cutoff value. Instead, the objective of the titration algorithm was to identify the individualized MAP corresponding to the lowest reproducible RRI, which we hypothesized reflects a hemodynamic state with relatively lower intrarenal vascular impedance.

Second, we acknowledge that the original manuscript inaccurately linked the 0.02 threshold to observer variability. We have corrected this description. The $\Delta$RRI $\geqslant$ 0.02 threshold was derived from repeatability testing under the same blood pressure condition by the same examiner, where three consecutive RRI measurements were obtained at each time point. The observed short-term measurement fluctuation was approximately 0.02. Therefore, during titration, an RRI change $\geqslant$0.02 was predefined as exceeding expected measurement fluctuation and interpreted as a meaningful directional hemodynamic response rather than measurement noise. Importantly, this threshold was used as an operational rule during titration rather than as a disease-specific or prognostic cutoff. For transparency, the raw measurement data used for this repeatability analysis have been uploaded as supplementary material.

Third, the stepwise norepinephrine adjustment ($\pm$0.02 μg/kg/min) was selected as the smallest clinically practical dose change that can reliably produce a measurable change in MAP. In our pilot observations, this adjustment typically resulted in a MAP change of approximately 5–10 mmHg, which allowed gradual exploration of the MAP–RRI relationship while minimizing the risk of abrupt hemodynamic perturbation.

Fourth, the 5–10-minute reassessment interval was chosen to allow sufficient hemodynamic stabilization following each norepinephrine adjustment. Norepinephrine has a rapid onset of action (approximately 1–2 minutes) with near-steady hemodynamic effects occurring within several minutes; therefore, a 5–10-minute interval was considered adequate to observe the resulting RRI response.

Finally, the baseline MAP used to initiate the titration procedure was defined as the MAP measured after completion of early resuscitation and initiation of vasopressor therapy, consistent with the clinical stabilization phase described in current sepsis management guidelines.

These clarifications have now been incorporated into the revised Methods section to better explain the physiological and methodological considerations underlying the titration algorithm.

**Because the intervention is complex and treating clinicians were not blinded, intervention fidelity is a critical determinant of internal validity. However, the manuscript does not describe how compliance with the RRI-guided strategy was assessed. The authors should clarify how adherence was operationally defined, how it was measured, and provide quantitative compliance data for the intervention arm. Without such information, it is not possible to interpret the results properly.**

Response: We thank the reviewer for this important comment. We agree that intervention fidelity is critical in a complex, non-blinded hemodynamic intervention. We have now clarified in the revised manuscript how adherence to the RRI-guided strategy was operationally defined, measured, and quantified.

In the intervention arm, protocol adherence comprised two components:

(1) titration fidelity, defined as completion of the prespecified RRI-guided norepinephrine titration algorithm, with stepwise NE adjustments of ±0.02 μg/kg/min, corresponding MAP changes of 5–10 mmHg, a stabilization period of 5–10 minutes after each adjustment, and triplicate RRI measurements averaged at each pressure level, until the MAP associated with the lowest RRI was identified; and (2) maintenance fidelity, defined as maintaining MAP within ±5 mmHg of this individualized target MAP during the subsequent 12-hour intervention window. Quantitatively, 132/134 patients in the intervention arm met the prespecified criteria for a protocol-defined V-shaped MAP–RRI relationship, allowing individualized target MAP determination. In the remaining 2 patients, no clear inflection point was observed, and MAP was managed according to the prespecified fallback range of 65–70 mmHg. During the 12-hour maintenance phase, the mean time-in-target MAP was 89.7% (10.76 hours), indicating high adherence to the intervention protocol. We have added these details to the Methods and Results sections to improve interpretability of the intervention effect.

**The use of baseline MAP as an initial stratification variable raises additional concerns. In patients with septic shock, reliable baseline blood pressure values are frequently unavailable or difficult to define, particularly when vasopressors have already been initiated or when patients are transferred from other settings. The manuscript does not explain how missing or unreliable baseline MAP values were handled. The authors should specify the data source and time window used to define baseline MAP, the proportion of patients with missing data, and the prespecified approach to missingness or uncertainty.**

Response: We thank the reviewer for this important comment. We have clarified the definition and handling of baseline MAP in the revised manuscript.

Baseline MAP was defined as the real-time invasive arterial pressure recorded at the moment immediately prior to randomization, after completion of initial resuscitation and under stable vasopressor support. Because all patients were monitored via continuous invasive arterial catheter in our ICU, a real-time MAP value was available for every patient at the time of randomization. No averaging over a time window was applied; the MAP value at the point of randomization was used directly.

Regarding potential concerns about patients transferred from other settings: all enrolled patients were admitted directly to the ICU of our center, where initial resuscitation was completed and hemodynamic stability was confirmed before randomization. Patients transferred mid-resuscitation from other hospitals were not randomized until resuscitation was complete and vasopressor support was stable. Therefore, all baseline MAP values were obtained under standardized conditions within our ICU.

All 274 randomized patients had available baseline MAP data; no missing values required imputation. Baseline MAP was used for descriptive stratification only and did not determine the titration target, which was identified individually based on the MAP–RRI relationship during the intervention.

**The statistical analysis section does not clearly state whether the primary analysis was conducted according to the intention-to-treat principle or using a per-protocol approach. Please explicitly define the primary analysis population and describe how protocol deviations were handled.**

Response: We thank the reviewer for this important comment. We have clarified the analysis populations in the revised manuscript. The primary outcome (28-day mortality) was analyzed according to the intention-to-treat principle, including all 274 randomized patients. Secondary hemodynamic and organ-support outcomes were analyzed in patients who survived beyond 24 hours and underwent protocol-directed titration (n = 268), as these outcomes required exposure to the intervention. These distinctions are now clearly stated in the Methods and Results sections.

**There is an important inconsistency between the original protocol and the manuscript regarding sample size. The protocol reportedly planned enrollment of 120 patients, whereas the sample size calculation in the main text yields a required sample of 268 patients. This discrepancy should be transparently addressed, including when and why the target sample size was changed and whether this modification occurred before or after trial initiation.**

Response: We thank the reviewer for highlighting this important issue. We have clarified the timeline and rationale for the sample size modification in the revised manuscript.

The original protocol planned enrollment of 120 patients based on preliminary clinical estimates. By March 2023, all 120 participants had been enrolled. At that stage, an internal review of the observed event rates and recruitment feasibility was conducted to reassess the statistical power of the trial. This review was not designed as a formal efficacy interim analysis and no predefined stopping rules were specified.

Using the observed event rates (28-day mortality 10.0% vs 24.3%), a formal power calculation indicated that 268 patients (134 per group) would be required to achieve 80% power with a two-sided $\alpha$ of 0.05, assuming a 10% dropout rate. The protocol amendment to expand the target sample size was approved by the institutional ethics committee in March 2023, and enrollment under the revised target began on May 20, 2023. Because this review was not intended for early stopping for efficacy or harm, a formal DSMB was not involved in this decision.

The timeline and rationale for this amendment have now been clarified in the Methods section..

**Finally, the assumptions underlying the sample size calculation appear overly optimistic. The authors assume a 28-day mortality of 10% in the intervention group compared with 24.3% in the control group, implying a very large absolute risk reduction. Experience in critical care medicine suggests that even multifaceted interventions rarely achieve mortality reductions of this magnitude. The authors should justify this expectation with supporting quantitative data from prior studies or pilot work, or revise the assumptions to more plausible effect sizes.**

Response: We thank the reviewer for this important comment and agree that the assumed absolute risk reduction of 14.3% was ambitious. This effect size was not selected arbitrarily; rather, it was derived from a local pilot study conducted at our institution (n=60), in which the 28-day mortality was 24.3% in the control group and 10.0% in the intervention group. At the time the trial was designed, no prior randomized data on RRI-guided MAP titration in sepsis were

available, and this pilot dataset represented the only quantitative basis for estimating the treatment effect.

We agree, however, that a treatment effect estimated from a small single-center pilot study is prone to overestimation because of limited sample size, local practice patterns, and chance variation. We have therefore revised the manuscript to explicitly acknowledge that the original sample size assumptions were optimistic and that the present trial may have been underpowered to detect a smaller, more clinically plausible mortality benefit.

In the current trial, the observed 28-day mortality difference (15.6% vs. 25.3%) was smaller than the pilot-based estimate and did not reach statistical significance, which is consistent with the reviewer's concern. We have revised the Methods to clarify the source of the sample size assumptions and revised the Discussion to state more explicitly that the study should be interpreted as an exploratory trial with respect to mortality, and that future multicenter confirmatory trials should be powered using more conservative effect-size assumptions.

To further improve transparency, we have added the pilot study sample size and event rates to the Sample Size Calculation section and strengthened the limitation statement accordingly.

Minor comments

**In the abstract, "P < 0.05" should be replaced with the exact p-value.**

Response: We thank the reviewer for this comment. All instances of "P < 0.05" in the abstract have been replaced with exact P values. For comparisons where the statistical software reported P < 0.001, we have retained this notation.

**The method used to determine baseline serum creatinine should be described in detail, including data sources and time windows, as this definition strongly influences the incidence of acute kidney injury.**

Response: We thank the reviewer for this important comment. We have revised the Methods section (Outcomes subsection) to explicitly define baseline serum creatinine. The manuscript now states: "Baseline serum creatinine (SCr) was defined as the first value measured at ICU admission. All creatinine measurements were obtained from the hospital electronic medical record system." This clarification specifies the time point and data source used for AKI classification.

**When reporting norepinephrine doses, the authors should specify that doses refer to norepinephrine base rather than alternative salt formulations.**

Response: We thank the reviewer for this comment. All norepinephrine used in this study was norepinephrine bitartrate. All doses reported in the manuscript refer to the commercially available norepinephrine bitartrate formulation, consistent with clinical practice in China. Vasopressor exposure was expressed as norepinephrine equivalent dose (NED, mg/day).

**A recent randomized trial demonstrated increased mortality with routine elevation of MAP targets to 80–85 mmHg (https://link.springer.com/article/10.1007/s00134-025-07910-4), but this study is not cited. Comparison with that trial would strengthen the discussion and better**

**contextualize the present findings.**

Response: We have cited this trial as Reference [4] in the revised manuscript.

**The sentence “A critical physiological relationship exists between MAP and RRI I n sepsis” should be corrected to “A critical physiological relationship exists between MAP and RRI in sepsis.”**

Response: We thank the reviewer for identifying this typographical error. The sentence has been corrected in the revised manuscript.

**The limitations inherent to single-center trials should be discussed more explicitly, including the well-documented difficulty in replicating promising single-center results in larger multicenter studies. Citation of this manuscript would help clarify this issue (https://link.springer.com/article/10.1186/s13054-023-04755-5).**

Response: We thank the reviewer for this important suggestion. We have strengthened the first limitation to explicitly acknowledge the challenges in replicating promising single-center findings in multicenter trials and have added the suggested reference in the Discussion[36].

Reviewer #3 (Remarks to the Author):

Thank you for providing me the opportunity to review this manuscript. Wang and colleagues report a single-center RCT evaluating RRI–guided MAP titration in patients with sepsis requiring vasopressors. The study addresses an important and unresolved question in sepsis management which is individualization of blood pressure targets beyond a uniform MAP threshold. The physiological rationale is sound, and the trial demonstrates feasibility of bedside US–guided hemodynamic titration. The primary outcome was negative. Some secondary endpoints are positive, however, less so after cadjustment for multiplicity. several methodological and interpretative issues limit the strength of the conclusions. The findings should be considered hypothesis-generating rather than practice-changing.

I have some major comments for the authors

**The trial did not demonstrate a statistically significant reduction in 28-day all-cause mortality, which was the prespecified primary endpoint. Nevertheless, the abstract, discussion, and conclusion emphasize reductions in AKI stage 3, organ-support–free days, and arrhythmias, which may overstate the clinical impact of the intervention.The conclusions should be tempered to reflect that the primary endpoint was negative. Secondary and exploratory outcomes should be clearly framed as hypothesis-generating! and language suggesting clinical efficacy should be avoided.**

Response: We thank the reviewer for this important comment. We agree that the primary endpoint was not met. We have revised the Abstract, Discussion, and Conclusion to clearly state that no significant reduction in 28-day mortality was observed. Secondary outcomes are now explicitly described as exploratory and hypothesis-generating, and language suggesting definitive clinical efficacy has been removed.

**2. A few patients who died shortly after randomization were excluded from the analysis. Excluding patients after randomization—particularly those who die may introduce attrition bias and undermines randomization.I am missing a strict ITT analysis including all randomized patients. I think this should be performed and reported. Or at minimum, a sensitivity analysis including these patients. Please discuss implications of their exclusion.**

Response: We thank the reviewer for this important comment. We agree that excluding patients after randomization, particularly those who died early, may introduce attrition bias. We have therefore revised the analysis to strictly follow the intention-to-treat principle. All 274 randomized patients, including the six patients who died within 24 hours after randomization, are now included in the primary analysis and analyzed according to their original group allocation. The CONSORT flow diagram, Methods, Results, and Tables have been updated accordingly.

**3. The study was single-blinded, with treating clinicians aware of group assignment. Several secondary outcomes (ventilator-free days, CRRT-free days, ICU length of stay) are susceptible to clinician discretion and therefore to performance and detection bias. This should be explicitly acknowledged for these outcomes. Were any standardized criteria or blinded adjudication used for decisions regarding ventilation or CRRT discontinuation? Was this standardised=?**

Response: We thank the reviewer for this important comment. Our ICU operates under unified institutional quality-control standards. Ventilator weaning followed a standardized ICU protocol based on daily readiness assessment and spontaneous breathing trials, consistent with the ATS/ACCP clinical practice guideline for liberation from mechanical ventilation (Am J Respir Crit Care Med, 2017).

Decisions regarding CRRT initiation and discontinuation were made by the attending intensivist but followed predefined institutional criteria aligned with KDIGO recommendations (Kidney Int Suppl, 2012), including hemodynamic stability, recovery of urine output, and adequate metabolic control. These protocols were applied uniformly to both study groups regardless of treatment allocation.

No blinded adjudication committee was used for ventilator or CRRT decisions. We have clarified this in the Methods and acknowledged the potential for performance bias for clinician-dependent outcomes in the Discussion.

**4. The control group maintained a mean MAP of approx 80 mmHg, which exceeds the Surviving Sepsis Campaign recommendation of ≥65 mmHg. To me, this means: The study compares two relatively high-MAP strategies rather than RRI-guided management versus guideline-based care. Please comment on this and reflect this in the Discuussion. rationale for this higher MAP in the control group should be clarified. I suggest to discuss how this affects generalizability and interpretation of the findings.**

Response: We thank the reviewer for this important comment. We acknowledge that the control group maintained a mean MAP of approximately 80 mmHg, which exceeds the minimum target recommended by the Surviving Sepsis Campaign. This likely reflects local bedside practice rather than protocol-mandated MAP escalation. We have clarified in the Discussion that our study compares an individualized RRI-guided strategy with a relatively high-MAP standard care approach, which may affect generalizability to settings strictly adhering to lower MAP targets.

Because both groups maintained relatively high MAP levels, the observed benefits in the MTG are more likely attributable to the individualization of MAP targets based on the MAP–RRI relationship rather than to the absolute MAP level achieved.

**5. RRI measurement is very operator dependent, yet all ultrasound assessments were performed by two trained examiners at a single center. No quantitative inter-observer or intra-observer reliability data are provided. Can you provide these? Otherwise, this needs be acknowledged as a limitation.**

Response: Thank you for highlighting the importance of measurement reliability for Doppler-derived RRI. To evaluate inter-observer variability, a reproducibility analysis was conducted using paired RRI measurements obtained independently by two trained examiners prior to the initiation of the study.

A total of 20 patients (40 RRI values) were randomly selected for duplicate measurements. The mean absolute difference between observers was 0.011, with a mean bias of −0.001. Bland–Altman analysis demonstrated 95% limits of agreement ranging from −0.029 to 0.027, indicating good inter-observer agreement.

These results are consistent with prior methodological literature on Doppler-derived RRI measurements and support the reliability of the ultrasound assessments performed in this study. We have now added these data to the Methods and Results sections of the revised manuscript.

**6. The intervention lasted only 12 hours, whereas hard outcomes (mortality!) were assessed up to 28 days. The causal link between a brief hemodynamic intervention and long-term survival,renal or respiratory outcomes remains uncertain.**

Response: We thank the reviewer for this important comment. We agree that the causal link between a 12-hour hemodynamic intervention and 28-day clinical outcomes cannot be established from this trial and warrants careful interpretation. We have acknowledged this as a limitation in the Discussion. Importantly, several lines of evidence support the biological plausibility that early hemodynamic optimization may influence downstream outcomes: (1) RRI measured on the first day of ICU admission has been shown to predict AKI development at 3–5 days[16,17]; (2) early goal-directed resuscitation within the first 6 hours of sepsis is associated with improved prognosis[7]; and (3) early renal perfusion injury may initiate inflammatory cascades that affect later organ function. Nonetheless, we acknowledge that the 12-hour intervention window is a significant limitation, and the observed associations with 28-day outcomes should be interpreted as hypothesis-generating. Whether sustained or repeated RRI-guided titration over longer periods would yield stronger effects remains to be investigated in future trials.

**7. Numerous secondary and subgroup analyses were performed. After conservative correction methods (Bonferroni), only CRRT-free days remain statistically significant. Emphasis on findings that remain significant only under FDR correction is quite speculative.. I suggest thatsecondary outcomes should be clearly framed as exploratory. avoid drawing strong conclusions from results that are sensitive to the method of multiple-comparison adjustment.**

Response: We thank the reviewer for this important comment. We agree that the multiple

secondary and subgroup analyses increase the risk of type I error. In the revised manuscript, we have clarified that all secondary outcomes are exploratory and have revised the Results and Discussion sections to interpret these findings cautiously. Specifically, we now note that only CRRT-free days remained statistically significant under the conservative Bonferroni correction, and the secondary findings should be considered hypothesis-generating rather than confirmatory.

8. **Subgroup analyses by hypertension status and baseline AKI were performed, but these analyses appear underpowered and are at risk of false-positive findings. --> I would suggest deleting these or discussing them appropriately as only mechanistc and exploratory.**
Response: We thank the reviewer for this thoughtful suggestion. We have taken a two-part approach to address this concern:
First, the “Renal Autoregulation Assessment” subsection—which comprised within-arm observational comparisons of MAP, RRI, and clinical outcomes between hypertensive vs. non-hypertensive patients and between AKI vs. non-AKI patients within the MTG only—has been removed. These analyses were mechanistic in nature and did not address treatment-effect modification, and their inclusion in the main text could be perceived as data-dredging.
Second, the prespecified between-arm subgroup analyses (MTG vs. CTG stratified by hypertension and baseline AKI status) have been retained in the main text but are now extensively reframed. The subsection is now titled “Exploratory Subgroup Analyses” and begins with an explicit statement that these analyses were not powered for subgroup comparisons. All findings are described as hypothesis-generating. We believe this approach balances the reviewer’s concern about overinterpretation with the value of preserving prespecified subgroup data for transparency and hypothesis generation, in keeping with CONSORT guidance on subgroup reporting.

Minor Comments

**1. To my mind, the manuscript would benefit from clearer adherence to CONSORT guidelines reporting.. especially more detailed reporting of allocation concealment and randomization implementation.**
Response: We thank the reviewer for this important comment. In the revised manuscript, we have clarified the randomization procedures in accordance with CONSORT recommendations. Specifically, the random allocation sequence was generated using computer-based random number generation. Participants were then assigned to study groups according to this predefined randomization sequence. Outcome assessors responsible for data analysis were blinded to group allocation.

**2. On several occasions, the authors equate lowest RRI with optimal renal autoregulation. However, RRI is influenced by multiple factors (e.g., pulse pressure, arterial compliance, venous congestion, et cetera) and does not directly measure renal blood flow!**
Response: We thank the reviewer for this important clarification. We conducted a systematic review of the entire manuscript and identified 12 instances in which RRI was inappropriately equated with renal autoregulatory function or direct renal blood flow measurement. All have been revised. Specifically: (1) All references to RRI as a “direct measure” of renal vascular

resistance have been replaced with language describing RRI as a composite Doppler-derived surrogate index; (2) The nadir of the MAP – RRI curve is now described as corresponding to "a hemodynamic state associated with lower intrarenal vascular impedance" rather than "optimal autoregulation"; (3) We have added a dedicated paragraph in the Discussion acknowledging the multifactorial determinants of RRI (pulse pressure, arterial compliance, venous congestion) and the limitation that RRI cannot directly identify the lower limit of autoregulation; (4) The Results subsection previously titled "Renal Autoregulation Assessment" has been renamed to avoid the implication that RRI directly assesses autoregulatory function.

**3. CEUS data are presented but appear to be available only in a subset of patients, and the clinical relevance of the observed changes is unclear? Please elaborate and clarify**

Response: We thank the reviewer for this important comment. We agree that the presentation of CEUS data in the original manuscript was insufficiently clear regarding the subset design, the nature of the statistical comparisons, and the clinical interpretation of the findings. We have addressed each of these concerns with the following revisions: First, we have specified the CEUS subset sample size. CEUS assessments were performed in a convenience subset of patients in whom logistical conditions permitted (MTG, n=81; CTG, n=71). The Methods section now explicitly states this subset design and provides the exact number of patients assessed.Second, we have clarified the nature of the CEUS comparisons. The primary CEUS analysis was a within-group (pre – vs. post-titration) comparison in the MTG, which demonstrated significant increases in peak intensity (pi; P = 0.031) and area under the time – intensity curve (AUC; P = 0.034) after RRI-guided MAP titration.

**4. the manuscript is lengthy and contains repeated physiological explanations. I would suggest shortening it**

Response: We have substantially revised the Introduction and Discussion by identifying and eliminating redundant physiological explanations. Specifically: (1) Repeated descriptions of organ perfusion determinants (CO, MAP, vascular resistance) were retained in the Introduction and removed from the Discussion; (2) The SEPSISPAM trial findings, discussed three times across both sections, are now introduced once and referenced briefly thereafter; (3) Extended mechanistic descriptions (NO/ROS/endothelin pathways) were condensed; (4) The paragraph defining "titration" was removed as self-evident. These changes reduced the combined word count of the Introduction and Discussion by approximately 500 words (from ~1,570 to ~1,070 words).

REVIEWER COMMENTS

Reviewer #1 (Remarks to the Author):

The authors have provided a thoughtful and constructive response to all my comments, which dealt with the statistical analyses, presentation of results and interpretation, of their clinical trial. Quantitative results are presented with all relevant details when reported in the text.

**Table 1 . Omissions have been corrected. The table now stands as a detailed report without the need to refer to a large number of footnotes.**

Response: We thank the reviewer for this positive and encouraging comment. We have further refined Table 1 to improve clarity and readability, ensuring that key variables are clearly presented within the table itself. Abbreviations have been minimized and organized in a concise footnote, including only essential definitions, to avoid unnecessary complexity and reliance on extensive footnotes.

**Table 2. and Figure 3. Further detail for results have been added.**

**The article is now focused on the main objective, the reporting of the primary outcome. The sensitivity analyses and the results for a large number of exploratory outcomes which added little and were often difficult to interpret have been omitted.**

Response: We thank the reviewer for this positive comment. Table 2 now presents the primary outcome with the complete effect estimate, 95% confidence interval, and exact P value. The Kaplan–Meier survival curve (Figure 3) is retained to provide visual representation of the survival distribution.

**Introduction**

**Please ensure all acronyms defined at first use (PSV, EDV on page 2 is not defined until page 4).**

Response: We thank the reviewer for this helpful comment. We have revised the Introduction to ensure that all abbreviations, including peak systolic velocity (PSV) and end-diastolic velocity (EDV), are defined at first use.

**Secondary outcomes - AKI stage**

**The incidence of stage 3 AKI at 28 days is reported "Compared to the CTG, the MTG had a lower incidence of stage 3 AKI at 28 days (18/134 vs. 33/134 RR, 0.545 (95%CI, 0.324-0.919; P=0.020))" But there is no explanation that this is stage 3 AKI compared with stage1 plus stage 2 AKI plus no AKI. (This is also reported in the abstract without a clear explanation).It would be preferable and clearer to either analyse and report either the total incidence of AKI compared with no AKI, or the assortment of the patients between the 3 stages of AKI and no AKI. Table 3 The incidence of AKI is correctly analysed and reported (there is no statistically significant difference between the two groups) but the further analysis of AKI is incorrect. The 4 classes AKI stage1, AKI stage2, AKIstage3 and no AKI are not independent. The correct analysis is a CHI2 (3) for assortment between the 4 classes within the 2 treatment groups. (CHI2 (3) = 6.88, p=0.08). There is no statistically significant difference in how the patients are assorted between the 4 classes.**

Response:We thank the reviewer for these two closely related comments, which we address together.

We agree that the four AKI categories (no AKI, stage 1, stage 2, and stage 3) are not independent, and that the appropriate analysis is a chi-squared test with 3 degrees of freedom comparing the distribution of patients across these four mutually exclusive categories between the two treatment groups.

We have revised the manuscript accordingly:

First, the overall incidence of any AKI versus no AKI is reported: MTG 64/134 (47.8%) versus CTG 71/134 (53.0%); RR, 0.901 (95% CI, 0.710 – 1.144); P = 0.392, with no significant difference between groups. The reviewer has confirmed that this analysis is correctly reported.

Second, each patient was classified by the highest KDIGO stage reached during the 28-day follow-up, yielding four mutually exclusive categories (no AKI, stage 1, stage 2, stage 3). The overall distribution across these categories is now presented in Table 3 with the chi-squared test as the primary analysis ( $\chi^2$(3) = 6.88, P = 0.076) (The reviewer cited p=0.08, which corresponds to our reported value of P = 0.076 prior to rounding.), confirming no statistically significant difference between groups. Stage-specific incidences with risk ratios and 95% confidence intervals are retained in Table 3 for descriptive purposes only. Stage-specific P-values have been removed to avoid misleading interpretation of non-independent comparisons.

Third, given that the overall distribution test did not reach statistical significance, we have removed all language describing a significant reduction in stage 3 AKI from the Abstract, Results, Discussion, and Conclusion. The previously reported stage 3 AKI finding is no longer presented as an independent positive result. AKI stage 3 has also been removed from the multiple comparison correction table (Table S6), which now includes three secondary endpoints with updated correction factors. Figure 4 has been revised to remove individual AKI stage comparisons.

**Figure 4 the data showing individual stages of AKI should be removed.**

Response: We thank the reviewer for this important comment. In the revised manuscript, Figure 4 has been modified to remove the individual AKI stage comparisons in order to avoid potential misinterpretation, as AKI staging represents a non-independent categorical variable.

Reviewer #2 (Remarks to the Author):

After reading the reply letter and revised manuscript, I still have several concerns.

**1. In Table 3, stage 3 AKI occurred in 18 patients of the intervention arm but CRRT was performed in 20 patients. Given that receipt of CRRT is considered stage 3 AKI according to the KDIGO guidelines, the authors should clarify this inconsistency.**

Response: We thank the reviewer for this important comment. In our study, CRRT initiation and discontinuation followed predefined institutional criteria that were applied uniformly to both treatment groups. As detailed in the Supplementary Material, CRRT was initiated when one or more of the following conditions were present: anuria or severe oliguria, hyperkalemia with electrocardiographic changes, severe metabolic acidosis (pH < 7.2), blood urea nitrogen > 84 mg/dL, serum creatinine > 5.65 mg/dL (499 μmol/L), or refractory fluid overload.

Therefore, CRRT initiation in our cohort was based on urgent clinical indications and was not restricted to patients who had already met serum creatinine- or urine output-based stage 3 thresholds at the time of CRRT initiation. We acknowledge that this analytical approach differs from incorporating CRRT receipt directly into categorical stage 3 AKI classification under the full

KDIGO framework. For the categorical AKI stage analysis in this manuscript, patients were classified according to serum creatinine and urine output criteria, whereas CRRT use was analyzed separately as an organ-support variable. This explains why the number of patients receiving CRRT does not exactly match the number classified as stage 3 AKI in Table 3. We have now clarified this point in the Methods.

**2. Use of the serum creatinine value at ICU admission as the baseline value typically underestimates the rate of AKI properly since in septic patients such values are already elevated compared to the true baseline value.**

Response: We thank the reviewer for this important comment. We agree that using the serum creatinine value at ICU admission as the baseline likely underestimates the true incidence of AKI, as creatinine may already be elevated at the time of ICU admission in septic patients compared with their premorbid values.

We acknowledge this as a limitation. However, premorbid creatinine values were not available for the majority of our patients, as many were admitted directly to the ICU without recent outpatient laboratory data. Using the ICU admission creatinine was therefore a pragmatic decision to ensure a uniform and reproducible baseline across all patients. This approach is consistent with KDIGO recommendations, which suggest using the first available value as baseline when premorbid creatinine is unknown.

Importantly, this limitation applies equally to both treatment groups due to randomization, and is therefore unlikely to introduce differential bias in the between-group comparison of AKI outcomes. Nevertheless, the absolute incidence of AKI in both groups may be underestimated, and our findings should be interpreted accordingly.

We have added this point to the Limitations section of the revised manuscript.

**Reviewer #3 (Remarks to the Author):**

**The authors adequately responded to my remarks and I think the manuscript's quality is strongly improved. The authors should be appalauded for this study and I recommend publishing it.**

Response: We sincerely thank the reviewer for this positive and encouraging comment. We greatly appreciate the reviewer’s recognition of our efforts to improve the manuscript.

*"Renal Resistive Index-Guided Mean Arterial Pressure Titration in Sepsis: A Prospective Randomized Controlled Trial"*

***Reviewer #1 Comment:*** *The authors have provided a detailed and considered response to my final comments concerning the results for the AKI grading. These are now reported accurately and with clarity. I have only reviewed statistical analyses and results and I recommend publishing this study which has been carried out to a high standard.*

**Response:** We are deeply grateful to Reviewer #1 for the thorough review of the statistical analyses and for recommending publication.

***Reviewer #2 Comment:*** *The authors have appropriately addressed my previous comments. I have no further comments.*

**Response:** We sincerely thank Reviewer #2 for the constructive feedback throughout the review process and for confirming that all previous comments have been satisfactorily addressed.

We thank the editor and both reviewers once again for their thoughtful and constructive engagement with our work. We have addressed all editorial requests and confirm that the revised manuscript, supplementary information, source data, figures, CONSORT checklist, English-translated protocol, completed Reporting Summary, and Author Checklist have been prepared in accordance with Nature Communications guidelines.

We hope the revised manuscript is now suitable for publication and look forward to your decision.

Sincerely,

**Lixia Liu**
